# Copy Number Variations of CEP63, FOSL2 and PAQR6 Serve as Novel Signatures for the Prognosis of Bladder Cancer

**DOI:** 10.3389/fonc.2021.674933

**Published:** 2021-05-10

**Authors:** Zhao Cai, Huang Chen, Jingqiao Bai, Yang Zheng, Jianhui Ma, Xiongwei Cai, Yu Liu, Kaitai Zhang, Jianzhong Shou, Yanning Gao

**Affiliations:** ^1^ State Key Laboratory of Molecular Oncology, Department of Etiology and Carcinogenesis, National Cancer Center/National Clinical Research Center for Cancer/Cancer Hospital, Chinese Academy of Medical Sciences and Peking Union Medical College, Beijing, China; ^2^ Department of Pathology, China-Japan Friendship Hospital, Beijing, China; ^3^ Department of Urology, National Cancer Center/National Clinical Research Center for Cancer/Cancer Hospital, Chinese Academy of Medical Sciences and Peking Union Medical College, Beijing, China

**Keywords:** copy number variation, bladder cancer, prognosis, CEP63, FOSL2, PAQR6

## Abstract

**Background:**

Finding effective prognostic signatures is of great urgency due to the high risk of recurrence and progression of bladder cancer (BC). Although a lot of genetic alterations are involved in the carcinogenesis, none of them were referred in the current risk group stratifications. In this study, we aimed to find significant copy number variations (CNVs) to predict prognosis for BC patients.

**Methods:**

CNVs with high aberration frequencies in BC were explored by array-based comparative genomic hybridization in 65 tumor samples. Candidates were validated in independent groups of BC tumor samples (n=219) and urine samples (n=123). 3D digital PCR was applied for detecting accurate gene copy numbers in BC urine. In order to explore the prognostic value of candidate CNVs, all enrolled patients were followed up for the disease-free survival (DFS). Cox proportional hazards regression analysis was performed to find the independent prognostic factors for DFS.

**Results:**

CNVs of CEP63, FOSL2 and PAQR6 with high aberration frequencies (67.7%, 56.9% and 60.0%, respectively) were found in BC tumors. Copy numbers of CEP63, FOSL2 and PAQR6 were gained in 219 tumor samples. CNVs of CEP63 and FOSL2 were correlated with advanced tumor stage and high grade. Retrospective analysis (median follow-up time: 69 months) revealed that CNVs of CEP63 and FOSL2 were independent prognostic factors for DFS of non-muscle-invasive bladder cancer (NMIBC) patients, while CNVs of FOSL2 and PAQR6 were independent prognostic factors for DFS of muscle-invasive bladder cancer (MIBC) patients. Models for predicting DFS were constructed based on CNVs of three genes. Patients with high prognostic indexes tended to have poor DFS. Prognostic index can also help to identify those with worse outcomes among high risk NMIBC patients. Copy number gains of CEP63 and FOSL2 in urine were found to be significantly correlated with poor DFS of NMIBC patients.

**Conclusions:**

CNVs of CEP63, FOSL2 and PAQR6 were capable of predicting DFS and may serve as promising signatures for prognosis of BC.

## Introduction

Bladder cancer (BC) is the most common malignancy of the urinary tract, with approximately 550,000 newly diagnosed cases per year worldwide (1). Most BCs are urothelial carcinomas (2). Urothelial bladder cancer is classified into muscle-invasive bladder cancer (MIBC) and non-muscle-invasive bladder cancer (NMIBC) depending on whether the tumor invaded muscularis propria (3). Nearly 75% of BCs are NMIBCs (3). The standard treatment for NMIBC is transurethral resection of bladder tumor (TURBT), along with intravesical instillation of chemotherapy or vaccine-based therapy, followed by regular cystoscopy (4). The 5 year rates of tumor recurrence ranged from 50% to 70%, while the 5 year rates of progression ranged from 10% to 30% in NMIBC patients (3). It is reported (5) that high risk NMIBC patients (high grade Ta tumors, T1 tumors, carcinoma *in situ* or multiple, recurrent, and large (>3 cm) low grade Ta tumors) had a risk of 78% in recurrence and a risk of 45% in progression over 5 years. Those patients at high risk required correct treatment decision and should be paid more attention to on their surveillance. However, therapy strategies for high risk BC patients were still in debate. The prognosis prediction can be of great help for clinical decision making on treatment and surveillance practices. Approximately 25% of BCs are MIBCs (6), including those with distant metastasis, regularly treated by radical cystectomy, chemotherapy or radiotherapy (7). Approximately 50% of MIBC patients develop distant metastases, despite having received the standard therapy of radical cystectomy with pelvic lymph node dissection (PLND) (8).

The high recurrence nature of bladder cancer has urged researchers to pursue an efficient prognostic predictor for evaluating the risk. Despite the fact that current histopathologic classification has given rise to improvement in clinical management, evaluating the risk of recurrence still remains a challenge. Although various urinary markers based on different kinds of technologies have been recently developed, such as nuclear matrix protein-22 (NMP-22), bladder tumor antigen (BTA)-Stat, BTA-TRAK, and DD23 (9–11), none of these have been accepted as prognostic indicators in clinical guidelines, as their utility in predicting the prognosis of individual patients is not clear yet (4). However, evaluating recurrence or progression risk and assessing prognosis is critical since a delay in therapy can be life-threatening, especially for high-grade NMIBC and MIBC patients (12). Therefore, novel prognostic signatures, which are both effective for prognosis prediction and convenient for detection, are in urgent need.

Variations in gene copy number are commonly regarded as a source of difference in the genome sequences across individuals (13). As an initiative factor of genetic evolution and phenotypic variation, copy number variations (CNVs) present at the rate of 4.8%-9.5% (13) of the variability in the human genome. Other than benign polymorphic variants, CNVs are known to be associated with malignancies. Extra or missing copies of protein-coding genes or regulatory regions alter gene dosage and are related to various types of cancers (14).

BC, known for its high recurrence and metastasis rate, is characterized by a large number of genetic alterations involved in tumor development (15). However, the current risk group stratifications were still depending on clinical parameters such as recurrence history, tumor size, tumor number, histologic type, grade and stage (4, 7, 9), without any cancer related genetic alterations included.

**Figure 1:**
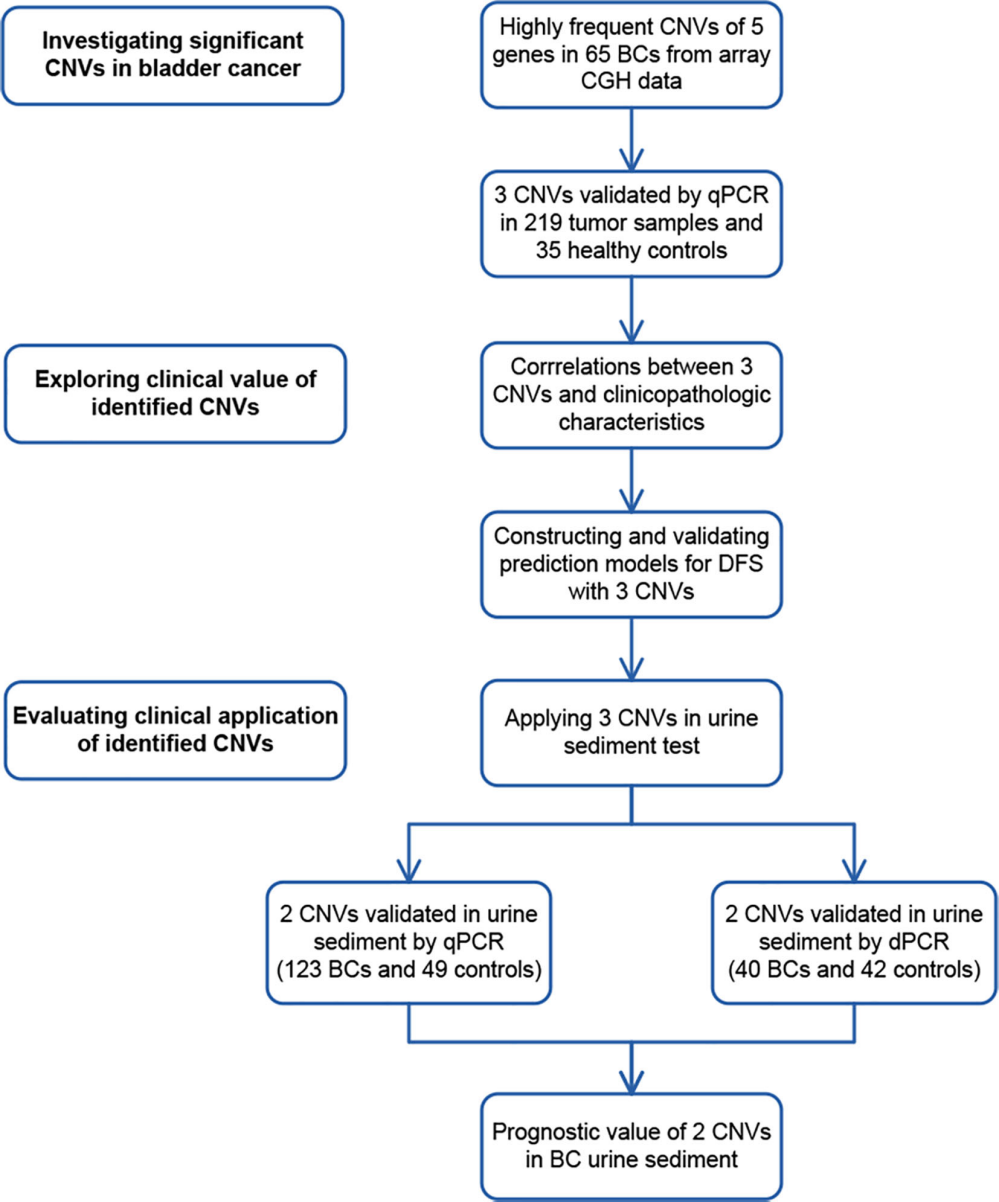
Study design. To discover significant copy number variations (CNVs), CNVs of 5 genes with high aberration frequencies were selected from array comparative genomic hybridization (CGH) data. Copy number gains of 3 genes were found in tumor samples by real-time PCR (qPCR). To explore their clinical value, correlations between the CNVs of 3 genes and clinicopathological characteristics were analyzed. CNVs of 3 genes were later applied in constructing and validating prediction models in 219 bladder cancer (BC) cases. To evaluate their potential clinical application, CNVs of 2 genes were validated by real-time PCR in 123 BC urine sediment samples and 49 healthy control samples. They were also validated by digital PCR (dPCR) in 40 BC urine and 42 healthy control samples. The prognostic value of 2 CNVs was further assessed in 123 urine samples.

In this study, we explored the CNVs with high aberration frequencies (>50%) from array-based comparative genomic hybridization (array CGH) data and validated in an independent group of 342 BC samples (including 219 tumor samples and 123 urine samples) by real-time polymerase chain reaction (PCR). 5 CNVs with high aberration frequencies were selected to explore their prognostic value in BC (**Figure 1**). The retrospective analysis of disease-free survival (DFS) was performed on all enrolled patients. Since there is a small amount of tumor cell DNA in urine, we adopted the highly sensitive 3D digital PCR to detect candidate CNVs in urine samples, to provide a simple and fast method for detection in clinical practice.

## Materials and Methods

### Patients and Samples

This study (CH-BMS-013) was approved by the Cancer Hospital, Chinese Academy of Medical Sciences Clinical Research Ethics Committee. Informed consent was obtained from all participants.

320 BC patients who were admitted to Cancer Hospital, Chinese Academy of Medical Sciences from July 1998 to March 2012 were enrolled in this study.

The inclusion criteria were as follows. (1) Patients were newly diagnosed and received treatment in Cancer Hospital, Chinese Academy of Medical Sciences. (2) The histopathological diagnoses of tumor were urothelial carcinoma of the bladder. The exclusion criteria were as follows. (1) Patients who received any neoadjuvant treatment before surgery were excluded. (2) Patients who had upper tract urothelial carcinoma or a history of upper tract urothelial carcinoma were excluded. (3) Patients who had a known history of other malignancy were excluded.

Tumor samples were staged and graded by a urological pathologist referring to the International Union Against Cancer (UICC) 2002 TNM staging system (16) and the World Health Organization (WHO) 2004 classification system (17), according to which carcinoma *in situ* and Ta–T1 stage was non-muscle-invasive cancer and T2–T4 stage was muscle-invasive cancer.

The tumor group included 219 cases with fresh-frozen tumor tissue samples. The urine group included 123 cases with preoperative urine sediment samples, among which 22 cases had both tissue and urine sediment samples. These 123 urine sediment samples were tested by real-time PCR. 40 of the 123 samples with DNA left after real-time PCR were further tested by 3D digital PCR. The clinicopathological characteristics of patients are outlined in **Table 1**.

**Table 1:** Clinicopathological characteristics of bladder cancer patients.

Characteristics	Tumor group (%)	Urine qPCR group (%)	Urine dPCR group (%)
	n=219	n=123	n=40
**Age (mean ± SD)**	60.7 ± 12.8	62.5 ± 12.4	55.1 ± 15.8
**Sex**			
Male	169 (77)	84 (68)	29 (73)
Female	50 (23)	39 (32)	11 (28)
**Smoking**			
No	129 (59)	83 (67)	26 (65)
Yes	90 (41)	40 (33)	14 (35)
**Drinking**			
No	164 (75)	97 (79)	32 (80)
Yes	55 (25)	26 (21)	8 (20)
**Tumor size**			
<3	114 (52)	98 (80)	30 (75)
≥3	105 (48)	25 (20)	10 (25)
**Number of tumors**			
Single	95 (43)	60 (49)	18 (45)
Multiple	124 (57)	63 (51)	22 (55)
**Stage**			
Ta+T1	127 (58)	104 (84)	21 (52)
≥T2	92 (42)	19 (16)	19 (48)
**Histologic grade**			
Low grade	77 (35)	78 (63)	20 (50)
High grade	142 (65)	45 (37)	20 (50)
**Lymph node metastasis**			
No	195 (89)	121 (98)	38 (95)
Yes	24 (11)	2 (2)	2 (5)
**Treatment**			
TURBT	63 (29)	100 (81)	27 (68)
Cystectomy	156 (71)	23 (19)	13 (32)

qPCR, real-time PCR; dPCR, digital PCR; SD, standard deviation; TURBT, transurethral resection of bladder tumor.

All 320 newly diagnosed patients were followed up for disease-free survival (DFS). The disease-free survival (DFS) in our study was defined as the period after a successful treatment during which there were no signs and symptoms of the recurrence or metastasis for NMIBC patients. For MIBC patients, the DFS was defined as the period after a successful treatment during which there were no signs and symptoms of the metastasis of tumor. For patients who had tumor recurrence or metastasis for many times, the DFS refers to the period before the first recurrence or metastasis.

35 blood samples from Chinese healthy donors were used as the control group for detecting CNVs of the 5 candidate genes in BC tissue. 91 urine sediment samples from donors without urinary tumors served as the control group for detecting CNVs in the urine.

Fresh-frozen tissue surgically resected from primary bladder tumors were obtained from patients who underwent standard treatment (4) including TURBT or cystectomy. The tissue was cleaned with saline and stored within 1 hour after removal at −80°C until use.

First morning urine samples of 200–500 ml were collected from BC patients on the date of surgery. The urine samples were centrifuged at 3000 × g for 20 minutes at 4°C and the sediments were stored at −80°C for DNA extraction. Blood samples of healthy donors were centrifuged to separate the plasma and blood cells within 1 hour after collection. Peripheral blood leukocytes were isolated using Erythrocyte Lysis Buffer (Qiagen, Germany) and frozen at –80°C until use.

### DNA Extraction

Fresh-frozen tissue and urine sediment DNA was extracted using QIAamp DNA Micro Kit #56304 (Qiagen, Germany). Blood sample DNA was extracted using DNeasy Blood & Tissue Kit #69506 (Qiagen, Germany). All procedures were performed according to the manufacturer’s protocol.

### Array-Based Comparative Genomic Hybridization

We performed array CGH on fresh-frozen tumor samples of 65 bladder cancer cases using human Genome CGH Microarray Kits, 4x44K #G4413A (Agilent Technologies, Santa Clara, CA, USA). All procedures were performed by the manufacturer’s instructions.

The chips were scanned on an Agilent G2565BA DNA Microarray Scanner. Image analysis was carried out by Feature-Extraction v.10.5.1.1 software (Agilent Technologies, Santa Clara, CA, USA). The array CGH data (GSE164743) had been uploaded on Gene Expression Omnibus (GEO).

### Real-Time Polymerase Chain Reaction (PCR)

Primers for real-time PCR were designed using Primer Premier 5.0 (Premier Biosoft International, Palo Alto, CA, USA). Primer sequences are shown in **Table 2**. Real-time PCR was performed using Mx3005p™ real-time PCR system (Agilent Technologies, Santa Clara, CA, USA). Each sample was tested in triplicate.

**Table 2:** Sequence of primers for real-time fluorescence quantitative PCR to detect the CNVs of candidate genes.

Target gene	Primer sequence (5’-3’)	Location	Product size (bp)
CEP63	Forward: CACTCGCTTTCCTCGGATTC	chr3:134,204,865-134,204,940	76 bp
	Reverse: CAATGCCTTCTCCAGACTTCC		
FOSL2	Forward: ACAGAGTGGAACAGCCGTATGC	chr2:28,637,135-28,637,350	216 bp
	Reverse: AAACCCAACTGCCCAATCTTCTTAG		
GHR	Forward : ATCCTTAGCAGAGCACCCT	chr5:42,629,151-42,629,308	158 bp
	Reverse: CCAGTTACTACCATCCCAAATA		
PAQR6	Forward: CCACACCTCAATCCACCAAACC	chr1:156,213,318-156,213,554	237 bp
	Reverse: CAGGGAAGAACTAACACGACTAACC		
ZFAND3	Forward: CACCTGATCAATTAAGAGGATTCGG	chr6:37,979,590-37,979,843	254 bp
	Reverse: GTGTGTGCTAAACATCTCAATTCTG		
TBP	Forward: GAACTGGCTTATAGGACTGT	chr6:170,865,900-170,866,078	179 bp
	Reverse: CTGGAACTCGTCTCACTATT		

Each reaction contained 20ng genomic DNA, 10μM paired primers, 0.5μl ROX Reference Dye II (50×) and 12.5μl SYBR^®^ Premix ExTaq™ (2×) (Takara Bio, Japan). After an initial denaturation step at 95°C for 10 sec, the amplifications were carried out with 45 cycles at a melting temperature of 95°C for 5 sec and an annealing temperature of 60°C for 20 sec. The specificity of the real-time PCR products was evaluated by melting curve analysis. TBP showed no variation in array CGH data and was therefore selected as the reference gene.

### 3D Digital PCR

3D digital PCR was performed using the QuantStudio™ 3D Digital PCR System (Applied Biosystems, Foster City, CA, USA). Copy numbers of target genes were detected using TaqMan^®^ Copy Number Assay and TaqMan^®^ Copy Number Reference Assay (Thermo Fisher Scientific, USA). Both assays were run simultaneously in a duplex PCR reaction.

14.5μl of reaction mixtures containing the 10ng DNA, 2×Mastermix, 20×Copy Number and 20×Reference Assay was loaded on the 3D Digital PCR Chip v2 and was performed PCR using the ProFlex™ 2x Flat PCR System. After an initial denaturation step at 96°C for 10min, the amplifications were carried out with 40 two-step cycles at 60°C for 2 min and 98°C for 30s and then an annealing step at 60°C for 2 min.

After PCR amplification, the chip was read by QuantStudio™ 3D Digital PCR Instrument. The fluorescent signals were further analyzed by QuantStudio™ 3D AnalysisSuite™ software.

### Data Analysis

Agilent Genomic Workbench 7.0 (Agilent, Santa Clara, CA, USA) was used for analyzing array CGH data. The Aberration Detection Method 1 (ADM-1) algorithm was adopted to identify DNA CNVs. The ADM-1 algorithm can identify the aberrant intervals with consistently high or low log-ratios based on the statistical score. A threshold of 6.0 was used according to the instruction manual. An aberration filtering option of a minimum of 3 probes in each region and the absolute average log_2_ ratio>0.25 was applied. Diploid centralization and the GC correction algorithm were used for data normalization. The University of California Santa Cruz (UCSC) human genome assembly hg19 was used as a reference. The genes with high aberration frequencies (>50%) were validated by subsequent real-time PCR. The copy number ratios relative to TBP of the candidate genes detected by real-time PCR were calculated using the comparative cycle threshold (Ct) 2^−ΔΔCt^ method.

Raw fluorescence data of 3D Digital PCR Chip v2 was read by the 3D Digital PCR Instrument. The ratio of FAM : VIC (ratio_FAM/VIC_) representing the adjusted copy number of target genes was calculated using the Poisson distribution by the QuantStudio™ 3D AnalysisSuite™ software (**Supplementary Figure 1**). To maximize the detection of copy number imbalance, ratios of candidate gene/TBP (ratio_candidate/TBP_) were calculated indirectly by dividing candidate gene ratio_FAM/VIC_ by TBP ratio_FAM/VIC_.

Statistical analysis was conducted using SPSS Statistics 25.0 (IBM Corp., Armonk, NY, USA). A p-value <0.05 on a two-sided test was considered significant. The Kolmogorov-Smirnov test was used to assess the normality of the data distribution. The group differences of continuous variables were assessed using the non-parametric Mann–Whitney U test (for non-normal distributions) and Student’s t-test (for normal distributions). Pearson’s χ^2^-test was used for categorical values. Bar graphs were drawn by GraphPad Prism 5 (San Diego, USA).

The cumulative incidence of copy number ratios and DFS, stratified by tumor stage, was plotted using Kaplan–Meier curves on “R” computing environment version 3. 6. 0 (18) (R Foundation for Statistical Computing, Vienna, Austria). The R package “survminer” was used to determine the optimal cutoff point for copy number ratios of identified genes in the survival analysis. The log-rank test was applied to assess the differences in DFS. Hazard ratios (HRs) for tumor recurrence or progression were calculated using univariate and multivariate Cox proportional hazards regression analysis with the forward logistic regression method. The proportional hazards assumption was satisfied (p>0.05) for variables in the Cox regression analysis for NMIBC and MIBC.

## Results

### Discovering Significant CNVs in BC

#### Investigating CNVs With High Frequencies From Array CGH Profiling

The array CGH data of 65 BC tumor samples (GSE164743) were used to explore CNVs with high aberration frequencies. A total of 108 gained fragments (length>1kb) and 279 lost fragments (length>1kb) were found in tumor samples (log_2_ ratio>0.25, **Supplementary Tables 1, 2**). 5 genes located on 5 fragments with high aberration frequencies (>50%) were selected as candidate genes for validation. The 5 genes were CEP63, FOSL2, GHR, PAQR6 and ZFAND3 (**Table 3**). Gains of copy numbers was found in 4 genes (CEP63, GHR, PAQR6 and FOSL2). Deletion of copy numbers was found in 1 gene (ZFAND3). TBP, with no copy number change in the array CGH data, was selected as an internal reference gene for subsequent real-time PCR validation.

**Table 3:** Candidate genes selected from array CGH profiling for validation.

Gene	Agilent probe ID	Location	Copy number	Frequency
CEP63	A_14_P100520	3q22.2	Gain	44/65
GHR	A_14_P127533	5p13-p12	Gain	43/65
PAQR6	A_14_P131501	1q22	Gain	39/65
FOSL2	A_14_P109117	2p23.3	Gain	37/65
ZFAND3	A_14_P102497	6pter-22.3	Loss	40/65
TBP	A_14_P114920	6q27	No change	0/65

#### Real-Time PCR Validation for CNVs of Candidate Genes

**Figure 2:**
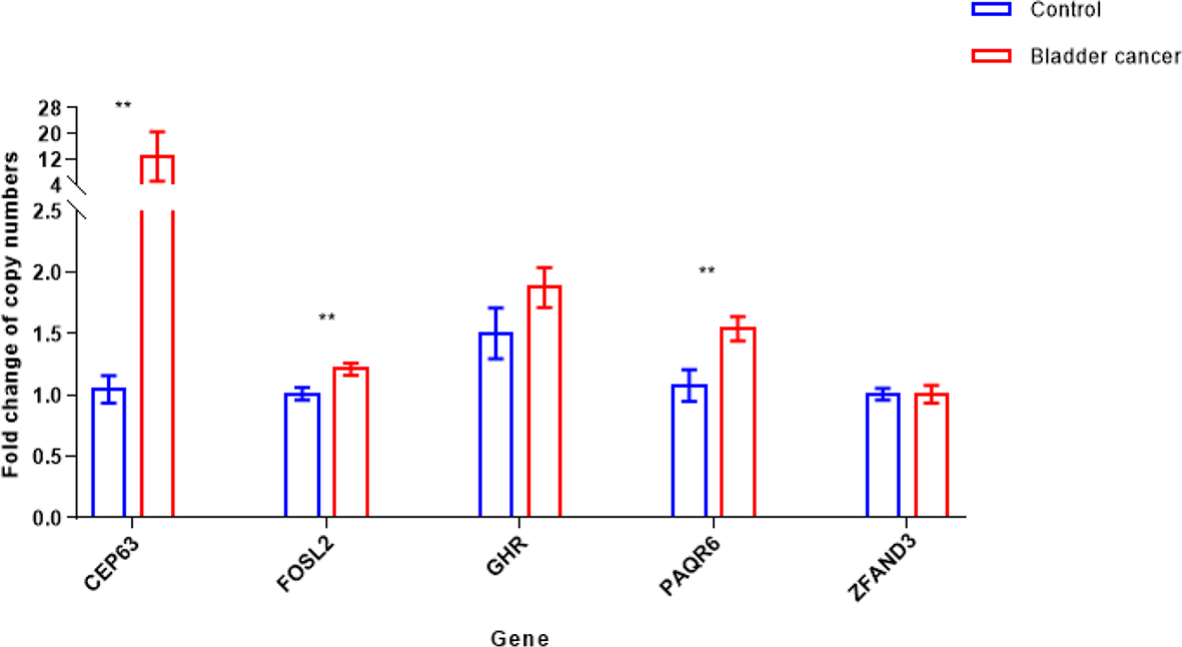
Copy number variations [mean with 95% confidence interval (CI)] of 5 candidate genes in bladder cancer tumor and control samples (**p < 0.01). Copy numbers of CEP63, FOSL2 and PAQR6 were significantly gained in 219 tumor tissue samples, determined by Mann-Whitney U test.

We performed real-time PCR on 219 fresh-frozen tumor tissue samples to validate CNVs of 5 candidate genes selected from the array CGH data. Peripheral blood samples from 35 healthy donors were used as the control. Results showed that compared with the control group, copy numbers of 3 genes, CEP63 (p<0.01), FOSL2 (p<0.01) and PAQR6 (p<0.01) were significantly gained in tumor tissues (**Figure 2**).

### Analyzing the Clinical Value of Identified Candidate Gene CNVs

#### Correlations Between Identified CNVs and Clinicopathological Characteristics

To evaluate the clinical significance of CEP63, FOSL2 and PAQR6 CNVs, we analyzed the correlations between CNVs of 3 genes and the clinicopathological characteristics of the 219 BC patients (**Table 4**). Among the 219 cases, there were 127 NMIBC cases and 92 MIBC cases. The copy numbers of CEP63 and FOSL2 were significantly different (p<0.01 and p<0.01, respectively) between the NMIBC and MIBC groups. Copy number gains of CEP63 and FOSL2 were correlated with advanced tumor stage and high tumor grade (p<0.01 and p=0.047, respectively). Copy number gains of CEP63 were also associated with positive lymph node metastasis (p<0.01). Copy numbers of PAQR6 were gained in BC but had no significant correlations with clinicopathological characteristics. No significant correlations between the CNVs of 3 genes and age, sex were found.

**Table 4:** Correlation between 3 CNVs and clinicopathological characteristics of 219 BC patients [median (interquartile range)].

Parameters	BC tumor (n=219)						
	Cases	CEP63	p value	FOSL2	p value	PAQR6	p value
**Age**			0.751		0.163		0.366
≤ 65	133	2.030 (1.755-4.227)		1.089 (0.973-1.267)		1.388 (1.052-1.861)	
> 65	86	2.181 (1.731-3.332)		1.125 (0.982-1.525)		1.489 (1.053-1.914)	
**Sex**			0.640		0.539		0.608
Male	169	2.077 (1.742-4.146)		1.109 (0.976-1.349)		1.433 (1.052-1.909)	
Female	50	2.040 (1.719-3.446)		1.089 (0.981-1.258)		1.393 (1.072-1.733)	
**Stage**			**1.475E-04**		**0.001**		0.821
Ta–T1	127	1.952 (1.664-2.831)		1.074 (0.950-1.186)		1.397 (1.112-1.793)	
T2–T4	92	2.327 (1.881-10.846)		1.173 (1.020-1.502)		1.447 (1.041-1.911)	
**Histologic grade**			**1.927E-06**		**0.047**		0.641
Low grade	77	1.784 (1.624-2.354)		1.069 (0.974-1.181)		1.388 (1.116-1.749)	
High grade	142	2.326 (1.881-6.038)		1.138 (0.980-1.461)		1.445 (1.049-1.918)	
**Lymph node metastasis**			**3.772E-07**		0.627		0.581
No	195	2.021 (1.715-3.084)		1.099 (0.977-1.338)		1.433 (1.086-1.878)	
Yes	24	13.790 (2.365-67.525)		1.158 (0.962-1.36)		1.307 (0.800-2.201)	

In bold: p < 0.05.

#### Correlations Between Identified CNVs and Disease-Free Survival (DFS)

To explore the prognostic value of 3 identified genes, CEP63, FOSL2 and PAQR6, all enrolled 320 patients were retrospectively analyzed for their DFS. The overall median follow-up time for 320 patients was 61 months, ranging from 3 to 188 months. The overall DFS for 320 patients at 5 and 10 years was 48.3% and 22.7%, respectively.

The median follow-up time for 219 patients with tumor samples was 69 months. 16 NMIBC patients and 16 MIBC patients were censored. The following survival analysis was stratified by NMIBC and MIBC. The median follow-up time for 111 NMIBC patients was 53 months, while the median follow-up time for 76 MIBC patients was 102 months.

**Figure 3:**
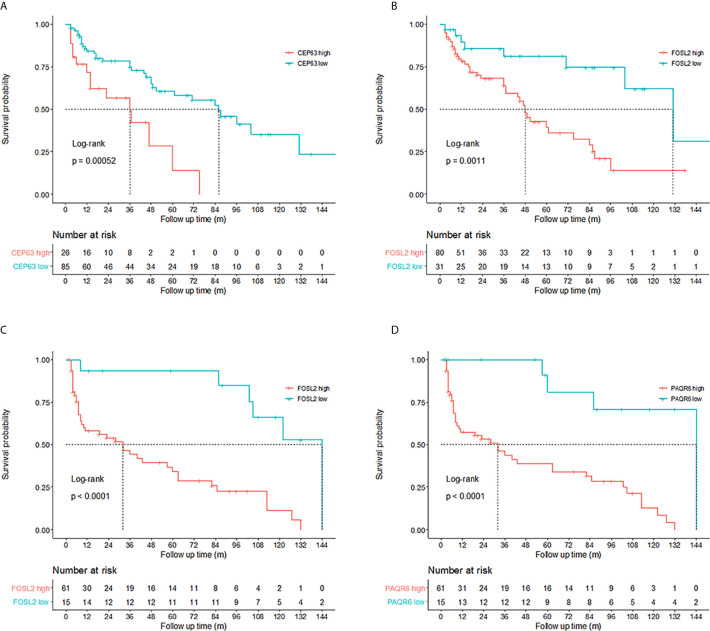
Prognosis prediction by 3 identified genes. Kaplan–Meier curves revealed that copy number gains of **(A)** CEP63 and **(B)** FOSL2 were related to poor disease-free survival (DFS) in non-muscle-invasive bladder cancer (NMIBC) patients (log-rank test, p<0.01). Cutoff points of copy number ratios relative to TATA-box binding protein (TBP) gene were 2.82 and 0.95 for CEP63 and FOSL2, respectively. Copy number gains of **(C)** FOSL2 and **(D)** PAQR6 were related to poor DFS in muscle-invasive bladder cancer (MIBC) patients (log-rank test, p<0.01). Cutoff points of copy number ratios of FOSL2 and PAQR6 were 0.99 and 0.78, respectively.

Kaplan–Meier tests were performed to analyze the correlations between the CNVs and DFS in 111 NMIBC and 76 MIBC patients. Results showed that NMIBC patients with copy number gains of CEP63 (copy number ratio>2.82) and FOSL2 (copy number ratio>0.95) had a significantly poorer DFS (p=0.00052 and p=0.0011, respectively, **Figure 3**). MIBC patients with copy number gains of FOSL2 (copy number ratio>0.99) and PAQR6 (copy number ratio>0.78) had a significantly poorer DFS (p<0.0001 and p<0.0001, respectively, **Figure 3**).

Univariate Cox proportional hazards regression analysis revealed the significant correlations between the DFS of NMIBC patients and CNVs of CEP63 (p=0.001) and FOSL2 (p=0.003). Multivariate analysis showed that CNVs of CEP63 (p=0.002) and FOSL2 (p=0.004) were independent prognostic factors for the DFS of NMIBC patients (**Table 5**). Significant correlations were found between the DFS of MIBC patients and CNVs of FOSL2 (p=0.0002) and PAQR6 (p=0.001) by univariate analysis. Multivariate analysis revealed that CNVs of FOSL2 (p=0.022) and PAQR6 (p=0.024) were independent prognostic factors for DFS of MIBC patients (**Table 5**).

**Table 5:** Univariate and multivariate Cox proportional hazard regression analysis for DFS prediction in NMIBC and MIBC patients.

NMIBC/MIBC	Parameters	Categories	Univariate analysis				Multivariate analysis			
			HR	95% CI		p value	HR	95% CI		p value
				lower****	****upper			lower	upper	
NMIBC	Age	>65 *vs.* ≤65	0.956	0.524	1.745	0.883				
	Sex	Male *vs.* Female	1.029	0.558	1.896	0.927				
	Tumor size (cm)	≥3 *vs.* <3	0.886	0.497	1.582	0.683				
	No. of tumors	Multiple *vs.* Single	1.321	0.735	2.376	0.352				
	Tumor stage	T1 *vs.* Ta	1.168	0.563	2.423	0.677				
	Histologic grade	High *vs.* Low	1.046	0.591	1.850	0.878				
	Lymph node metastasis	Positive *vs.* Negative	1.367	0.329	5.687	0.667				
	Surgery	RC *vs.* TURBT	0.510	0.181	1.436	0.202				
	Smoking	Yes *vs.* No	1.231	0.675	2.246	0.498				
	Drinking	Yes *vs.* No	1.890	0.951	3.753	0.069				
	CEP63 CNVs	High *vs.* Low	3.005	1.569	5.755	**0.001**	2.801	1.472	5.328	**0.002**
	FOSL2 CNVs	High *vs.* Low	3.303	1.503	7.258	**0.003**	3.146	1.430	6.921	**0.004**
	PAQR6 CNVs	High *vs.* Low	1.105	0.515	2.372	0.798				
MIBC	Age	>65 *vs.* ≤65	0.884	0.480	1.628	0.693				
	Sex	Male *vs.* Female	1.547	0.761	3.144	0.228				
	Tumor size(cm)	≥3 *vs.* <3	1.306	0.727	2.346	0.373				
	No. of tumors	Multiple *vs.* Single	1.279	0.707	2.314	0.416				
	Tumor stage	T3-4 *vs.* T2	1.365	0.756	2.467	0.302				
	Surgery	RC *vs.* PC	1.016	0.550	1.877	0.960				
	CEP63 CNVs	High *vs.* Low	0.709	0.395	1.275	0.251				
	FOSL2 CNVs	High *vs.* Low	6.130	2.345	16.006	**0.0002**	3.267	1.184	9.015	**0.022**
	PAQR6 CNVs	High *vs.* Low	8.060	2.436	26.692	**0.001**	4.300	1.209	15.301	**0.024**

DFS, disease-free survival; NMIBC, non-muscle-invasive bladder cancer; MIBC, muscle-invasive bladder cancer; CNVs, copy number variations; HR, hazard ratio; CI, confidence interval; RC, radical cystectomy; TURBT, transurethral resection of bladder tumor; PC, partial cystectomy. In bold: p < 0.05.

#### Prediction Models for the DFS of NMIBC and MIBC Patients

**Figure 4:**
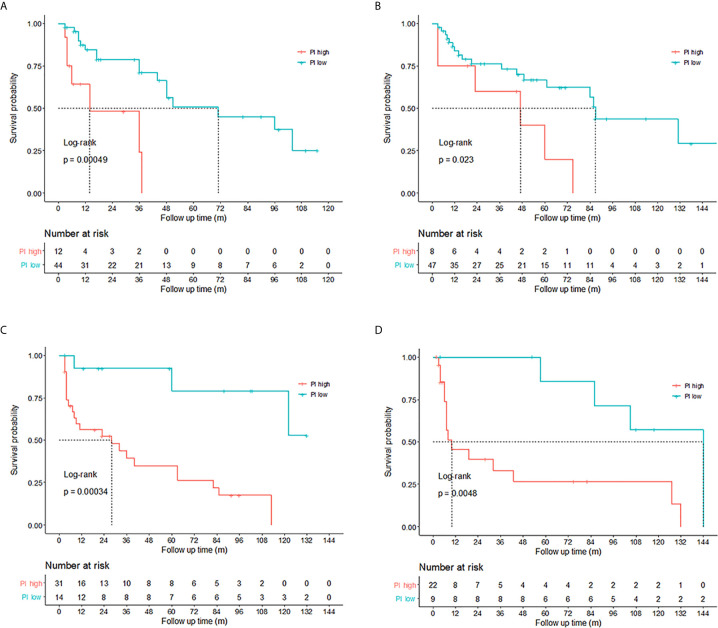
Validation of the prediction models for non-muscle-invasive bladder cancer (NMIBC) and muscle-invasive bladder cancer (MIBC). High prognostic index (PI) (PI>1.5095) indicated poor disease-free survival (DFS) (p<0.05) for NMIBC patients in the **(A)** training set (n=56) and **(B)** validation set (n=55). MIBC patients with PIs higher than 2.2079 tended to have poorer DFS (p<0.01) in the **(C)** training set (n=45) and **(D)** validation set (n=31). The differences between the high PI group and the low PI group were assessed by the log-rank test.

In order to construct prediction models for DFS, we randomly divided NMIBC and MIBC patients into training sets and test sets (**Table 6**). No differences were found between the training set and test set in terms of clinical parameters including age, sex, smoking, drinking, tumor size, tumor number, histologic grade and lymph node metastasis. Independent prognostic factors from multiple Cox regression analysis were used to construct prediction model equations in the NMIBC training set (n=56) and the MIBC training set (n=45). The prognostic index (PI) was applied for assessing the risk of recurrence for NMIBC and the risk of progression for MIBC. NMIBC and MIBC patients with high PIs had poorer DFS (p<0.05 and p<0.01, respectively) compared with those with low PIs (**Figure 4**).

**Table 6:** Clinicopathological characteristics of BC patients in the prediction model.

Characteristics	NMIBC		p value	MIBC		p value
	Training set	Validation set		Training set	Validation set	
	(n=56)	(n=55)		(n=45)	(n=31)	
**Age (mean ± SD)**			0.93			0.08
	59.3 ± 14.5	59.4 ± 12.3		60.7 ± 10.3	65.0 ± 10.6	
**Sex**			0.08			0.51^a^
Male	44	35		37	28	
Female	12	20		8	3	
**Smoking**			0.50			0.88
No	40	36		21	15	
Yes	16	19		24	16	
**Drinking**			0.67			0.92
No	44	45		30	21	
Yes	12	10		15	10	
**Tumor size (cm)**			0.29			0.68
<3	29	34		21	13	
≥3	27	21		24	18	
**No. of tumors**			0.38			0.98
Single	27	22		19	13	
Multiple	29	33		26	18	
**Stage**			0.51			–
Ta	13	10		–	–	
T1	43	45		–	–	
**Histologic grade**			0.30			1.00^a^
Low grade	30	24		6	4	
High grade	26	31		39	27	
**Lymph node metastasis**			0.62^a^			0.16
No	53	54		38	22	
Yes	3	1		7	9	

a Continuity correction of Pearson’s chi-square test.

SD, standard deviation.

The prediction model equation for NMIBC was as follows: copy number ratios of CEP63 were categorized as 1 if higher than the cutoff point of 2.82; otherwise, they were categorized as 0. Copy number ratios of FOSL2 were categorized as 1 or 0, using a cutoff point of 0.95. The p value of the likelihood ratio was 0.002.

$$\text{PI}=1.5095\times \text{CEP}63^{a}+1.47123\times \text{FOSL}2^{b}$$

Caopy number ratio of CEP63Cbopy number ratio of FOSL2

The prediction model equation for MIBC was as follows: copy number ratios of FOSL2 were categorized as 1 or 0, using a cutoff point of 0.99. Copy number ratios of PAQR6 were categorized as 1 or 0, using a cutoff point of 0.78. The p value of the likelihood ratio was 0.0001.

$$\text{PI}=2.0440\times \text{FOSL}2^{\text{a}}+2.2079\times \text{PAQR}6^{\text{b}}$$

Caopy number ratio of FOSL2Cbopy number ratio of PAQR6

The cutoff point of the PI was 1.5095 for NMIBC and 2.2079 for MIBC, which meant when the copy numbers of the 2 genes in the equation were both gained (copy number ratios higher than the cutoff points), the patient tended to have a poorer DFS. The prediction models were subsequently validated in the NMIBC validation set (n=55) and MIBC validation set (n=31). It was shown that NMIBC and MIBC patients with high PIs tended to have poor prognosis (p<0.01 and p<0.01, respectively, **Figure 4**).

#### Prognosis Prediction for High Risk NMIBC Patients

**Figure 5:**
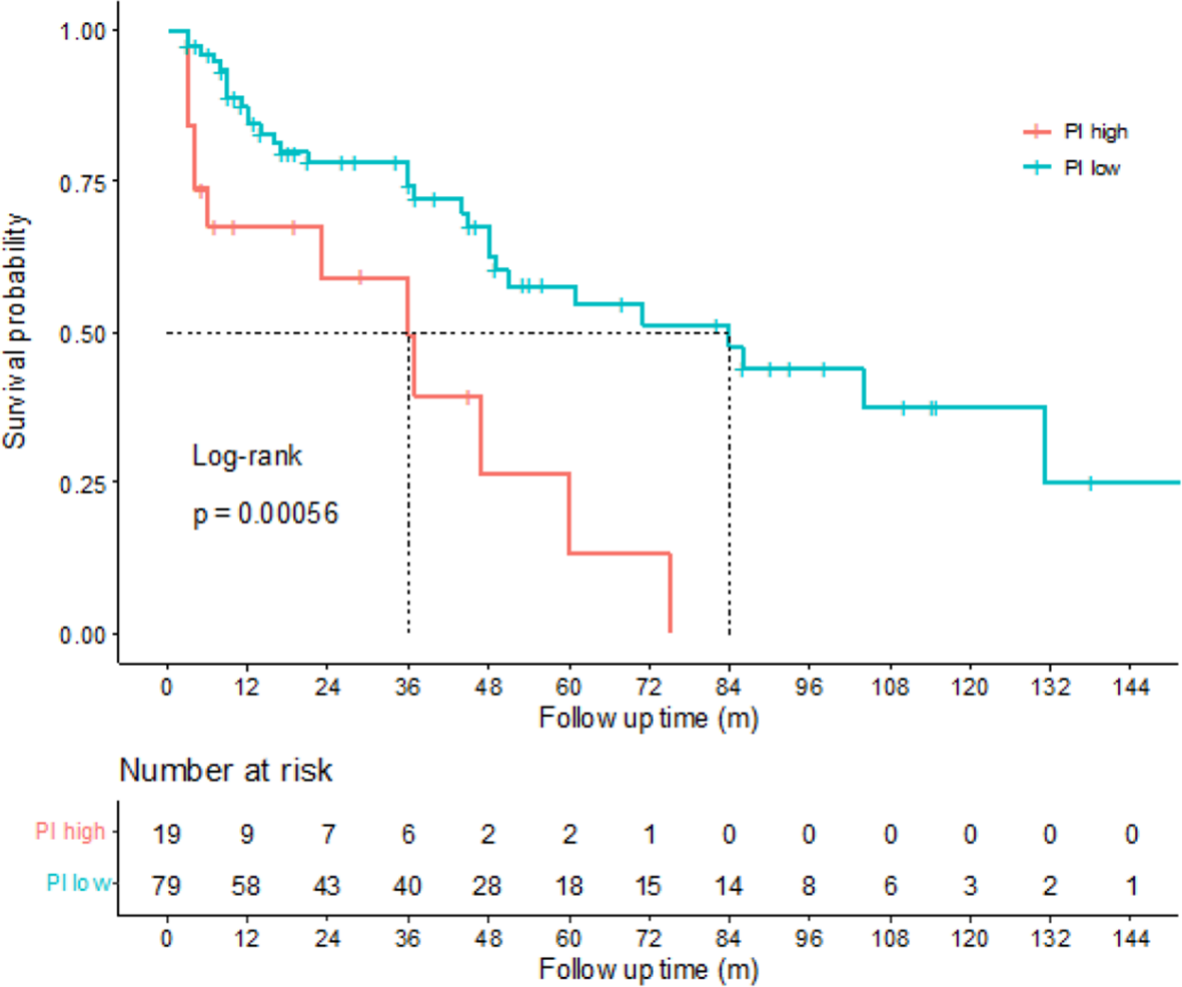
Correlations between disease-free survival (DFS) and prognostic index (PI) of high risk non-muscle-invasive bladder cancer (NMIBC) patients. Kaplan-Meier curves revealed that the high risk NMIBC patients with high PIs tended to have poorer DFS than the high risk NMIBC patients with low PIs (log-rank test, p=0.00056).

According to the risk group stratification in European Association of Urology Guidelines on NMIBC (19), patients with any of the following were at high risk: T1 tumor; G3 (high grade) tumor; carcinoma in situ, multiple, recurrent and large (>3 cm) Ta G1G2/low grade tumors (all features must be present). Among the 111 newly diagnosed NMIBC patients in our study, there were 98 patients at high risk and 13 patients at low or intermediate risk. Kaplan–Meier tests were performed to analyze the correlation between the PIs and DFS of 98 patients at high risk. The results showed that among the high risk NMIBC patients, those with high PIs had shorter DFS (p=0.00056), indicating that the PIs calculated by the prediction model can also be of help on predicting prognosis for high risk NMIBC patients (**Figure 5**).

Among the 111 NMIBC patients, DFS of 19 patients were less than 1 year, while DFS of 26 patients were more than 5 years. We analyzed the CNVs of CEP63 and FOSL2 in those patients and found that copy numbers of CEP63 (p=0.037) and FOSL2 (p=0.021) were significantly gained in NMIBC patients with DFS of less than 1 year compared with patients with DFS of more than 5 years (**Supplementary Figure 2**).

### Applying Identified CNVs to Clinical Practice

#### Detecting Identified CNVs in Urine Sediment Samples by Real-Time PCR

Currently, urinary tests are widely applied in clinical practice. Therefore, we tested the identified CNVs in 123 BC urine sediment samples to evaluate their clinical value for prognosis.

Real-time PCR results showed that compared with the 49 healthy control urine sediment samples, copy numbers of CEP63 (p=0.036) and FOSL2 (p<0.01) were significantly gained in BC urine sediment samples, which was consistent with the results in tumor samples (**Supplementary Figure 3**).

The correlations between the CNVs of 3 genes in urine and the clinicopathological characteristics of 123 BC patients were analyzed to assess the clinical significance of identified CNVs. However, no significant correlations were found between the identified CNVs in urine and clinicopathological characteristics (**Supplementary Table 3**).

#### Detecting Identified CNVs in Urine Sediment Samples Using 3D Digital PCR

The amount of tumor cell DNA in urine was much less than that in tumor tissues, thus making it difficult to obtain accurate gene copy numbers by real-time PCR. 3D digital PCR, a novel technique with high sensitivity, was therefore adopted for CNV detection in urine sediment samples to explore a promising method of urinary test.

3D digital PCR was conducted in 40 BC urine sediment samples with DNA left after tested by real-time PCR. Results showed that copy numbers of CEP63 (p<0.01) and FOSL2 (p<0.01) were significantly gained in 40 BC urine sediment samples compared with 42 control urine sediment samples (**Supplementary Figure 4**), which was consistent with the real-time PCR results of tumor samples and 123 urine sediment samples. Copy number gains of CEP63 (p<0.01) and FOSL2 (p=0.046) were found to be correlated with advanced tumor stage. CEP63 copy number gains (p=0.042) was also associated with high tumor grade (**Supplementary Table 4**). Those new findings may result from the improvement of sensitivity on detecting gene copy numbers by 3D digital PCR.

#### Prognostic Value of Identified CNVs in BC Urine Samples

In order to explore the prognostic value of the identified CNVs in urine sediment samples, we have retrospectively evaluated the 123 patients for DFS. 7 NMIBC patients were censored. The median follow-up time was 44 months.

**Figure 6:**
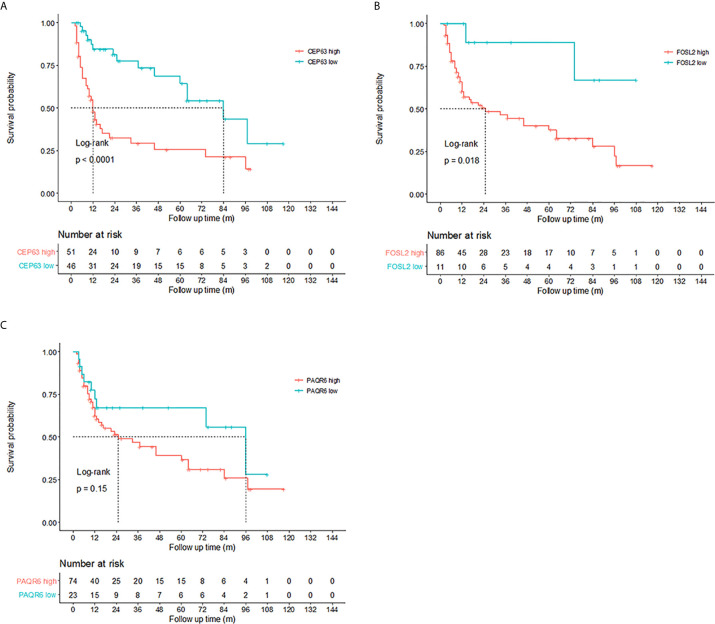
Prognosis prediction by 3 identified genes in urine sediment samples detected by real-time polymerase chain reaction (PCR). Kaplan-Meier curves showed that copy numbers gains of **(A)** CEP63 and **(B)** FOSL2 were associated with poor disease-free survival (DFS) in non-muscle-invasive bladder cancer (NMIBC) patients (p<0.0001 and p=0.018, respectively). The cutoff point of the copy number ratio relative to the TATA-box binding protein (TBP) gene for CEP63 and FOSL2 was 1.02 and 0.97, respectively. No correlations were found between DFS and CNVs of **(C)** PAQR6.

Kaplan–Meier tests were performed to analyze the correlations between the CNVs in urine and DFS of 97 NMIBC patients. Results showed that copy number gains of CEP63 (copy number ratio>1.02) and FOSL2 (copy number ratio>0.97) detected by real-time PCR were found to be significantly correlated (p<0.01 and p=0.018, respectively) with poor DFS of NMIBC patients (**Figure 6**), suggesting that CNVs of CEP63 and FOSL2 in urine can be the prognosis predictor for NMIBC patients.

## Discussion

In this study, we found that copy numbers of CEP63, FOSL2 and PAQR6 were significantly gained in BC. The array CGH data used for exploring candidate CNVs with high aberration frequencies were based on the patient cohort of the previous research. CNVs of CEP63 and FOSL2 were correlated with tumor stage and histologic grade. Copy number gains of CEP63 were also associated with lymph node metastasis. Survival analysis revealed a significant correlation between the CNVs of 3 identified genes and DFS. Copy number gains of CEP63 and FOSL2 were independent factors for DFS in NMIBC patients while copy number gains of FOSL2 and PAQR6 were independent factors for DFS in MIBC patients. The prediction models based on tumor samples were used for calculating PIs to predict the risk of recurrence for NMIBC and the risk of progression for MIBC patients. Among the high risk NMIBC patients, those who had high PIs tended to have poorer DFS than those with low PIs.

Since the role of 3 identified genes on prognosis prediction have been validated in tumor sample, they were further tested in urine samples for the convenience of application in clinical practice. Copy numbers of CEP63 and FOSL2 in urine showed significant correlations with poor DFS of NMIBC patients and were the significant predictors for prognosis. A highly sensitive and accurate technique, 3D digital PCR, was adopted to detect the exact gene copy numbers in urine. The consistency of the results with those in tumor samples suggested that this novel method may be helpful for urinary test in the future.

We have focused on identifying cancer related CNVs in this study, since unlike single-nucleotide polymorphisms, CNVs affect wider regions in the human genome and thus can cause imbalances in gene dosage with or without phenotypic consequences. A genome-wide CNV analysis of multiple cancer types have found that an average of 17% of the genome per tumor sample was amplified while 16% of it was deleted, indicating a much higher frequency of CNVs in malignant cells (20). Bioinformatics analysis of the GEO and TCGA public databases revealed some CNVs correlated with BC patient survival including 22 CNVs located on chr3p25 and chr11p11 (21) in MIBC and CCNE1 (22) in NMIBC. In a large population study, CNVs of GSTM1 and GSTT1 in blood samples were found to be related to survival in BC patients (23). However, these studies have not reported the correlations between BC and CNVs of the 3 identified genes in our study. Besides, there has not been any study on CNVs in BC combining mining from tumor tissues and applying in urine sediment for clinical use, which was assumed to produce more stable and reliable results. Furthermore, the sensitive 3D digital PCR method has not yet been adopted for detecting CNVs in BC urine samples.

The 3 identified genes we reported in this study have been reported in carcinogenesis in other cancers.

The CEP63-encoded centrosomal protein acts as a recruitment factor for cyclin-dependent kinase 1 (CDK1), which is essential for mitotic entry (24). Overexpression of CEP63 leads to *de novo* centrosome amplification and has been associated with poor prognosis in neuroblastoma patients (24). Elevated expression of CEP63 was also correlated with poor DFS of BC patients in TCGA Bladder Cancer (BLCA) data set (**Supplementary Figure 4**), which indicated the prognostic value of CEP63 on RNA levels. FOSL2 (FOS like 2, AP-1 transcription factor subunit) is a member of the Fos gene family. The FOS proteins have been implicated in a wide range of biological processes, including the regulation of cell proliferation, differentiation and transformation. Elevated expression of FOSL2 plays a key role in regulating the transforming growth factor beta (TGF-β) pathway (25). Abnormal expression of FOSL2 was also found in osteosarcoma (26), colon cancer (27) and ovarian carcinomas (28). According to our study, FOSL2 CNVs were independent prognostic factors for both NMIBC and MIBC, indicating their important role in tumor progression. Survival analysis of FOSL2 expression in the BLCA data set indicated a significant association between DFS and high expression of FOSL2, suggesting its prognostic value in BC (**Supplementary Figure 2**). PAQR6 is a member of the progestin and adipoQ receptor (PAQR) family (29). PAQR6 is coupled to G proteins and is a potential intermediary of the nonclassical antiapoptotic actions of neurosteroids in the central nervous system (30). Correlations were found between PAQR6 and tumor progression in endometrial cancer (31).

In this retrospective study, we have focused on the prognostic value of CEP63, FOSL2 and PAQR6 in bladder cancer. Carcinoma *in situ* was risk factor for tumor recurrence and progression. However, since it was much less common in NMIBC patients, there were not any CIS samples included in tumor group. We have verified the prognostic value of 2 CNVs in urine samples by 3D digital PCR. However, the sample size is a little small and only a few cases have both urine samples and tumor samples. Besides, an independent group of urine samples were needed for further validation. Apart from that, the reason why the CNVs of 3 genes were correlated with prognosis of BC patients needed to be illustrated by further research.

## Conclusions

In conclusion, we found that the cancer-related CNVs of CEP63, FOSL2 and PAQR6 were competent in evaluating recurrence or progression risk for BC patients and may be used for the risk group stratifications in the future. Copy number gains of CEP63 and FOSL2 in urine had the capability to be the novel urinary biomarkers for predicting recurrence risk. The innovative 3D digital PCR, applied in detecting CNVs in BC urine samples for the first time, may provide a new approach for urine-based surveillance of BC patients.

## Data Availability Statement

The original contributions presented in the study are included in the article/**Supplementary Material**. Further inquiries can be directed to the corresponding authors.****

## Ethics Statement

The studies involving human participants were reviewed and approved by the Cancer Hospital, Chinese Academy of Medical Sciences Clinical Research Ethics Committee under the study protocol number CH-BMS-013. The patients/participants provided their written informed consent to participate in this study.

## Author Contributions

Conceptualization, ZC, HC, JB and YZ. Methodology, JM, XC, and YL. Software, YL and XC. Formal analysis, ZC. Data curation, HC. Writing-original draft preparation, ZC. Writing-review and editing, ZC and HC. Supervision, KZ, JS and YG. Project administration, KZ, JS and YG. All authors contributed to the article and approved the submitted version.

## Funding

This work was supported by the Open Project of the State Key Laboratory of Molecular Oncology (SKL-KF-2013-02); Beijing Hope Run Special Fund of Cancer Foundation of China (LC2013A02); the Capital Characteristic Clinical Project (Z131107002213006).

## Conflict of Interest

The authors declare that the research was conducted in the absence of any commercial or financial relationships that could be construed as a potential conflict of interest.

## Supplementary Material

Supplementary Figure 1 3D digital PCR results of 5 candidate genes and 1 reference gene in BC and healthy control urine sediment. Gene copy numbers of 5 candidate genes (CEP63, FOSL2, GHR and PAQR6) and 1 reference gene (TBP) in (A) BC urine sediment and (B) healthy control urine sediment were analyzed by the QuantStudio™ 3D AnalysisSuite™ Software. Blue points near Y-axis of the plot indicated the sequences with only FAM reporter dye signal detected. Red points near X-axis of the plot indicated the sequences with only VIC reporter dye signal detected. Green points located midway between the FAM and VIC data point clusters in the plot suggested sequences with both FAM and VIC reporter dye signal. Yellow points located near the origin of the plot suggested sequences with no signal detected. A digital PCR Chip with more than 85% data point above threshold was applied for subsequent copy number analysis.

Supplementary Figure 2 Correlations between copy number variations (mean with 95% confidence interval [CI]) and disease-free survival (DFS) in non-muscle-invasive bladder cancer (NMIBC) patients (*p<0.05). Copy number variations of CEP63 (p=0.037) and FOSL2 (p=0.021) were significantly gained in NMIBC patients with DFS of less than 1 year, in comparison with patients with DFS of more than 5 years, determined by Mann-Whitney U test.

Supplementary Figure 3 Copy number variations (mean with 95% confidence interval [CI]) of 5 candidate genes in urine sediment detected by real-time PCR. Copy number variations of 5 candidate genes were tested in 123 bladder cancer urine and 49 healthy controls (*p<0.05, **p<0.01). Comparing with healthy controls, copy numbers of CEP63 (p=0.012) and FOSL2 (p=0.0001) were significantly gained in bladder cancer urine sediment, determined by Mann-Whitney U test.

Supplementary Figure 4 Copy number variations (mean with 95% confidence interval [CI]) of 5 candidate genes in urine sediment detected by digital PCR. Copy number variations of 5 candidate genes in 40 bladder cancer urine sediment and 42 healthy controls (**p<0.01) were detected by digital PCR. In comparison with control group, copy numbers of 2 genes (CEP63 and FOSL2) were significantly gained in BC urine sediment, assessed by Mann-Whitney U test.

Supplementary Figure 5 Recurrence prediction of CEP63 and FOSL2 expression in BLCA data set. Normalized BLCA gene expression RNAseq data (n=299) and clinical matrix were downloaded from UCSC Xena (https://xenabrowser.net). Kaplan-Meier curve revealed that high expression of (A) CEP63 and (B) FOSL2 were related to poor recurrence-free survival in 299 bladder cancer patients. Cutoff points of gene expression were 9.13 and 11.90 for CEP63 and FOSL2, respectively.

## Abbreviations

BC, bladder cancer; CNV, copy number variation; DFS, disease-free survival; MIBC, muscle-invasive bladder cancer; NMIBC, non-muscle-invasive bladder cancer; PCR, polymerase chain reaction.
