# Supplementary figures and images for: Copy Number Variations of CEP63, FOSL2 and PAQR6 Serve as Novel Signatures for the Prognosis of Bladder Cancer
Source: Front Oncol. 2021 May 10;11:674933. doi: 10.3389/fonc.2021.674933 (PMC8141655; doi:10.3389/fonc.2021.674933)

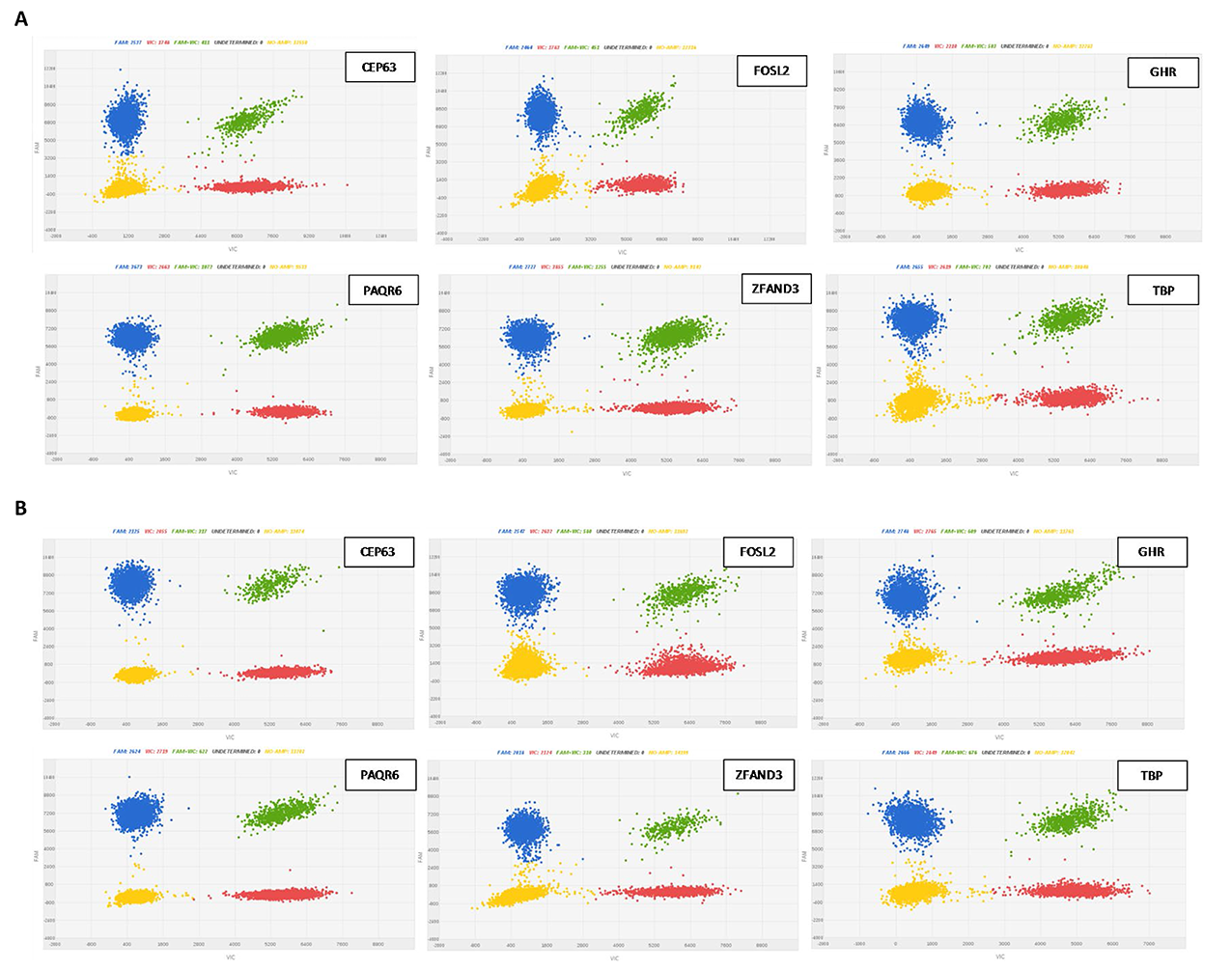

Supplement: Supplementary Figure 1 — 3D digital PCR results of 5 candidate genes and 1 reference gene in BC and healthy control urine sediment. Gene copy numbers of 5 candidate genes (CEP63, FOSL2, GHR and PAQR6) and 1 reference gene (TBP) in (A) BC urine sediment and (B) healthy control urine sediment were analyzed by the QuantStudio™ 3D AnalysisSuite™ Software. Blue points near Y-axis of the plot indicated the sequences with only FAM reporter dye signal detected. Red points near X-axis of the plot indicated the sequences with only VIC reporter dye signal detected. Green points located midway between the FAM and VIC data point clusters in the plot suggested sequences with both FAM and VIC reporter dye signal. Yellow points located near the origin of the plot suggested sequences with no signal detected. A digital PCR Chip with more than 85% data point above threshold was applied for subsequent copy number analysis. [file Image_1.tif]

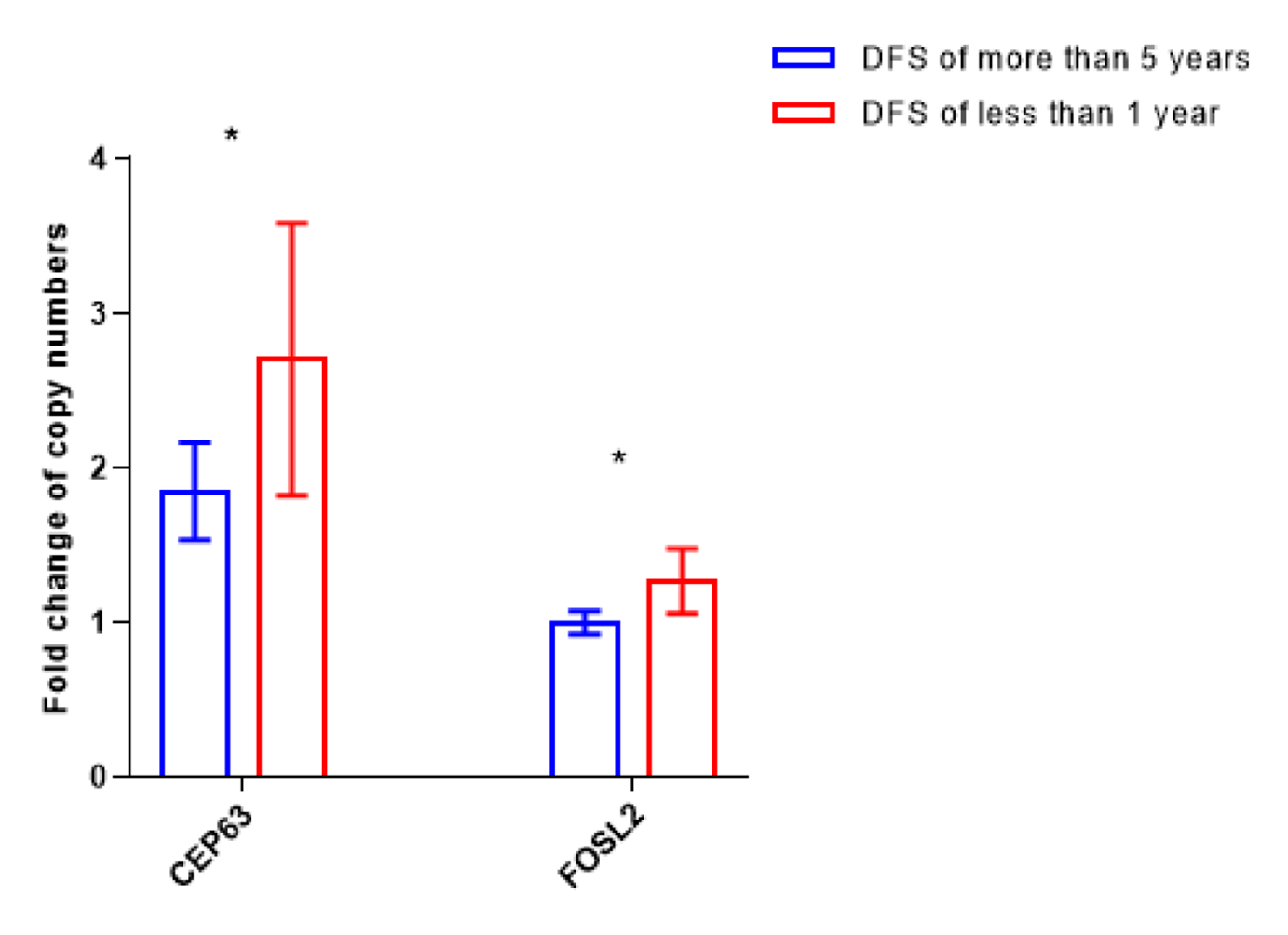

Supplement: Supplementary Figure 2 — Correlations between copy number variations (mean with 95% confidence interval [CI]) and disease-free survival (DFS) in non-muscle-invasive bladder cancer (NMIBC) patients (*p<0.05). Copy number variations of CEP63 (p=0.037) and FOSL2 (p=0.021) were significantly gained in NMIBC patients with DFS of less than 1 year, in comparison with patients with DFS of more than 5 years, determined by Mann-Whitney U test. [file Image_2.tif]

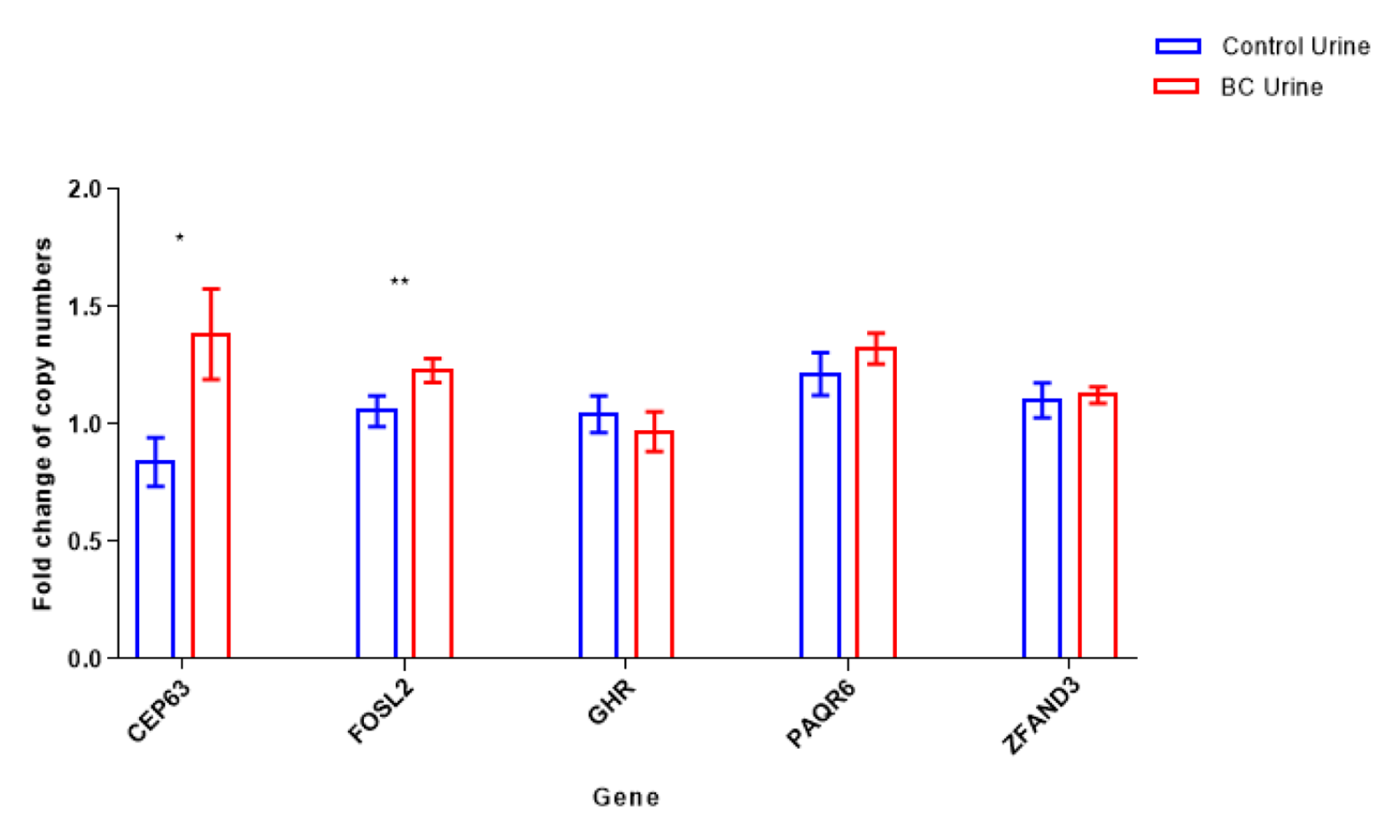

Supplement: Supplementary Figure 3 — Copy number variations (mean with 95% confidence interval [CI]) of 5 candidate genes in urine sediment detected by real-time PCR. Copy number variations of 5 candidate genes were tested in 123 bladder cancer urine and 49 healthy controls (*p<0.05, **p<0.01). Comparing with healthy controls, copy numbers of CEP63 (p=0.012) and FOSL2 (p=0.0001) were significantly gained in bladder cancer urine sediment, determined by Mann-Whitney U test. [file Image_3.tif]

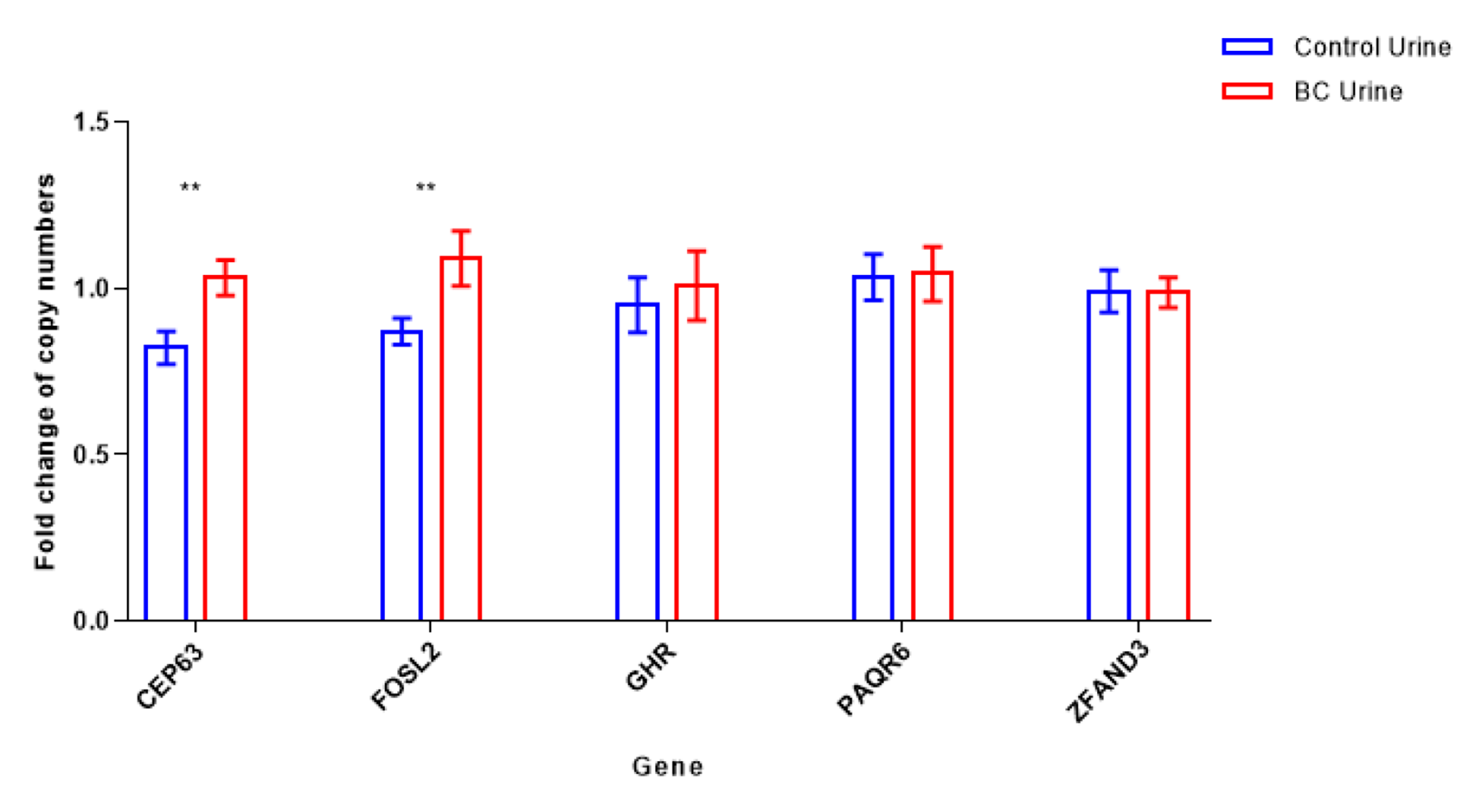

Supplement: Supplementary Figure 4 — Copy number variations (mean with 95% confidence interval [CI]) of 5 candidate genes in urine sediment detected by digital PCR. Copy number variations of 5 candidate genes in 40 bladder cancer urine sediment and 42 healthy controls (**p<0.01) were detected by digital PCR. In comparison with control group, copy numbers of 2 genes (CEP63 and FOSL2) were significantly gained in BC urine sediment, assessed by Mann-Whitney U test. [file Image_4.tif]

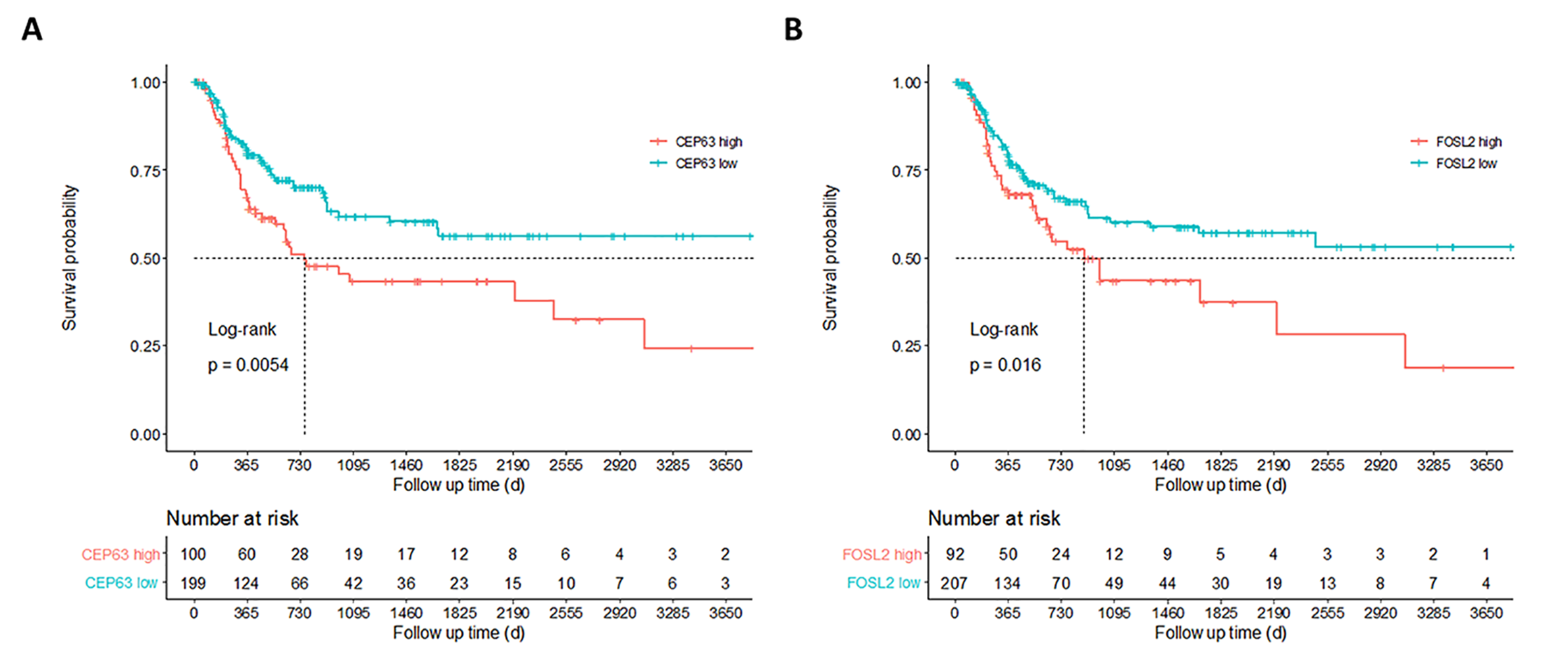

Supplement: Supplementary Figure 5 — Recurrence prediction of CEP63 and FOSL2 expression in BLCA data set. Normalized BLCA gene expression RNAseq data (n=299) and clinical matrix were downloaded from UCSC Xena (https://xenabrowser.net). Kaplan-Meier curve revealed that high expression of (A) CEP63 and (B) FOSL2 were related to poor recurrence-free survival in 299 bladder cancer patients. Cutoff points of gene expression were 9.13 and 11.90 for CEP63 and FOSL2, respectively. [file Image_5.tif]
