# Supplementary material for: Copy Number Variations of CEP63, FOSL2 and PAQR6 Serve as Novel Signatures for the Prognosis of Bladder Cancer
Source: Front Oncol. 2021 May 10;11:674933. doi: 10.3389/fonc.2021.674933 (PMC8141655; doi:10.3389/fonc.2021.674933)
Supplement: Supplementary file 6 [file Table_1.docx]

**Supplementary table 1.** Chromosome regions (length > 1kbp) frequently gained in 65 bladder tumors

| fragment | chromosome location | start (kb) | end (kb) | length (kb) |
| --- | --- | --- | --- | --- |
| 1 | 1q21.1 | 145580 | 145703 | 123 |
| 2 | 1q21.3 | 150354 | 150651 | 297 |
| 3 | 1q21.3 | 151026 | 151051 | 25 |
| 4 | 1q21.3 | 151224 | 151271 | 47 |
| 5 | 1q21.3 | 151629 | 151698 | 69 |
| 6 | 1q21.3 | 151901 | 151920 | 19 |
| 7 | 1q21.3 | 152511 | 152563 | 52 |
| 8 | 1q23.1 | 158431 | 158444 | 13 |
| 9 | 1q23.2 | 159399 | 159435 | 36 |
| 10 | 1q23.2 | 159458 | 159541 | 83 |
| 11 | 1q23.2 | 159749 | 159943 | 194 |
| 12 | 1q23.3 | 162799 | 163028 | 229 |
| 13 | 1q24.2 | 169441 | 169521 | 80 |
| 14 | 1q25.1 | 174699 | 174853 | 154 |
| 15 | 1q25.2 | 178408 | 178507 | 99 |
| 16 | 1q25.3 | 184911 | 184914 | 3 |
| 17 | 1q42.12 | 226669 | 226712 | 43 |
| 18 | 2q11.1 | 95347 | 95426 | 79 |
| 19 | 3q22.1 | 132474 | 132585 | 111 |
| 20 | 3q22.2 | 135656 | 135687 | 31 |
| 21 | 3q27.2 | 185587 | 185590 | 3 |
| 22 | 3q29 | 196653 | 196942 | 289 |
| 23 | 4p16.3 | 3465 | 3489 | 24 |
| 24 | 5p15.33 | 854 | 942 | 88 |
| 25 | 6p22.3 | 19703 | 19948 | 245 |
| 26 | 6p21.31 | 35311 | 35335 | 24 |
| 27 | 7p14.3 | 30971 | 30985 | 14 |
| 28 | 7p11.2 | 56304 | 56531 | 227 |
| 29 | 7q34 | 142671 | 142692 | 21 |
| 30 | 7q36.1 | 149161 | 149658 | 497 |
| 31 | 8q13.1 | 67126 | 67202 | 76 |
| 32 | 8q21.3 | 92107 | 92155 | 48 |
| 33 | 8q22.1 | 94807 | 94821 | 14 |
| 34 | 8q22.2 | 100025 | 100031 | 6 |
| 35 | 8q22.2 | 100563 | 100781 | 218 |
| 36 | 8q22.3 | 101699 | 102017 | 318 |
| 37 | 8q22.3 | 102283 | 102451 | 168 |
| 38 | 8q22.3 | 102680 | 103404 | 724 |
| 39 | 8q22.3 | 103633 | 103637 | 4 |
| 40 | 8q22.3 | 103931 | 104103 | 172 |
| 41 | 8q22.3 | 104406 | 104459 | 53 |
| 42 | 8q23.1 | 106883 | 107745 | 862 |
| 43 | 8q23.3 | 116779 | 117018 | 239 |
| 44 | 8q24.12 | 120005 | 120026 | 21 |
| 45 | 8q24.12 | 120418 | 120500 | 82 |
| 46 | 8q24.12 | 121526 | 121604 | 78 |
| 47 | 8q24.13 | 122722 | 123719 | 997 |
| 48 | 8q24.21 | 128818 | 128822 | 4 |
| 49 | 8q24.21 | 129574 | 130159 | 585 |
| 50 | 8q24.23 | 136554 | 136602 | 48 |
| 51 | 8q24.3 | 143739 | 143819 | 80 |
| 52 | 8q24.3 | 143954 | 143990 | 36 |
| 53 | 8q24.3 | 144311 | 144367 | 56 |
| 54 | 8q24.3 | 145062 | 145227 | 165 |
| 55 | 8q24.3 | 145464 | 145486 | 22 |
| 56 | 8q24.3 | 145612 | 145659 | 47 |
| 57 | 8q24.3 | 145693 | 145695 | 2 |
| 58 | 8q24.3 | 146201 | 146250 | 49 |
| 59 | 10q24.1 | 97984 | 98021 | 37 |
| 60 | 10q26.3 | 134895 | 134925 | 30 |
| 61 | 11q13.1 | 64905 | 65059 | 154 |
| 62 | 11q13.1 | 65168 | 65177 | 9 |
| 63 | 11q13.2 | 67109 | 67130 | 21 |
| 64 | 11q13.3 | 69165 | 69171 | 6 |
| 65 | 11q13.3 | 69333 | 69339 | 6 |
| 66 | 12p13.33 | 2526 | 2537 | 11 |
| 67 | 12q13.2 | 56431 | 56436 | 5 |
| 68 | 12q32.33 | 105028 | 105067 | 39 |
| 69 | 16p13.3 | 613 | 640 | 27 |
| 70 | 16p13.3 | 1735 | 1762 | 27 |
| 71 | 17p11.1 | 22879 | 22978 | 99 |
| 72 | 17q11.2 | 26841 | 26912 | 71 |
| 73 | 17q11.2 | 31441 | 31456 | 15 |
| 74 | 17q12 | 35076 | 35138 | 62 |
| 75 | 17q12 | 37518 | 37528 | 10 |
| 76 | 17q24.3 | 70367 | 70391 | 24 |
| 77 | 17q25.1 | 71355 | 71382 | 27 |
| 78 | 17q25.1 | 72998 | 73013 | 15 |
| 79 | 17q25.1 | 73645 | 73649 | 4 |
| 80 | 17q25.3 | 77449 | 77503 | 54 |
| 81 | 19p13.3 | 602 | 631 | 29 |
| 82 | 19p13.3 | 2180 | 2203 | 23 |
| 83 | 19q13.2 | 41289 | 41297 | 8 |
| 84 | 19q13.2 | 41333 | 41398 | 65 |
| 85 | 19q13.31 | 43437 | 43471 | 34 |
| 86 | 19q13.31 | 43563 | 43566 | 3 |
| 87 | 19q13.31 | 44113 | 44129 | 16 |
| 88 | 19q13.32 | 45804 | 45807 | 3 |
| 89 | 19q13.32 | 46727 | 46824 | 97 |
| 90 | 19q13.32 | 47443 | 47445 | 2 |
| 91 | 19q13.33 | 50174 | 50207 | 33 |
| 92 | 19q13.42 | 54818 | 54832 | 14 |
| 93 | 20p13 | 2975 | 3012 | 37 |
| 94 | 20p13 | 3729 | 3731 | 2 |
| 95 | 20q11.21 | 29536 | 29657 | 121 |
| 96 | 20q11.22 | 33342 | 33357 | 15 |
| 97 | 20q13.12 | 43949 | 43968 | 19 |
| 98 | 20q13.12 | 44034 | 44074 | 40 |
| 99 | 20q13.13 | 48633 | 48799 | 166 |
| 100 | 20q13.31 | 55368 | 55416 | 48 |
| 101 | 20q13.33 | 60225 | 60315 | 90 |
| 102 | 20q13.33 | 60553 | 60558 | 5 |
| 103 | 20q13.33 | 61382 | 61456 | 74 |
| 104 | 20q13.33 | 61633 | 61657 | 24 |
| 105 | 20q13.33 | 61747 | 61752 | 5 |
| 106 | 20q13.33 | 62134 | 62172 | 38 |
| 107 | 22q13.32 | 49291 | 49294 | 3 |
| 108 | 22q13.32-q13.33 | 49356 | 49525 | 169 |

**Supplementary table 2.** Chromosome regions frequently lost (length > 1kbp) in 65 bladder tumors

| fragment | chromosome location | start (kb) | end (kb) | length (kb) |
| --- | --- | --- | --- | --- |
| 1 | 2q22.3 | 147452 | 147651 | 199 |
| 2 | 2q23.3 | 152435 | 152504 | 69 |
| 3 | 2q24.3 | 166510 | 166601 | 91 |
| 4 | 2q33.1 | 202273 | 202385 | 112 |
| 5 | 2q33.3 | 206540 | 206629 | 89 |
| 6 | 2q35 | 216659 | 216750 | 91 |
| 7 | 2q36.1 | 223351 | 223572 | 221 |
| 8 | 2q36.3 | 230498 | 230576 | 78 |
| 9 | 2q37.1 | 231882 | 231907 | 25 |
| 10 | 2q37.3 | 240561 | 240571 | 10 |
| 11 | 3p24.2 | 25702 | 25735 | 33 |
| 12 | 4q34.1 | 173311 | 173585 | 274 |
| 13 | 4q35.2 | 189162 | 189249 | 87 |
| 14 | 5q11.2 | 56573 | 56596 | 23 |
| 15 | 5q11.2 | 58011 | 58055 | 44 |
| 16 | 5q12.1 | 61664 | 61694 | 30 |
| 17 | 5q13.2 | 68534 | 68591 | 57 |
| 18 | 5q13.2 | 68695 | 68698 | 3 |
| 19 | 5q13.2 | 68768 | 70784 | 2016 |
| 20 | 5q31.2 | 137520 | 137634 | 114 |
| 21 | 5q34 | 160961 | 161058 | 97 |
| 22 | 5q34 | 162813 | 162818 | 5 |
| 23 | 5q35.3 | 178164 | 178222 | 58 |
| 24 | 6p21.32 | 32605 | 32659 | 54 |
| 25 | 6q16.3 | 100749 | 100960 | 211 |
| 26 | 6q25.1 | 150088 | 150097 | 9 |
| 27 | 8p23.3 | 391 | 411 | 20 |
| 28 | 8p23.3 | 1422 | 1528 | 106 |
| 29 | 8p23.3 | 1642 | 1711 | 69 |
| 30 | 8p23.3-p23.2 | 2092 | 3127 | 1035 |
| 31 | 8p23.2 | 3313 | 3848 | 535 |
| 32 | 8p23.1 | 6281 | 6377 | 96 |
| 33 | 8p23.1 | 6572 | 6782 | 210 |
| 34 | 8p23.1 | 9410 | 9970 | 560 |
| 35 | 8p23.1 | 11258 | 11294 | 36 |
| 36 | 8p23.1 | 11604 | 11647 | 43 |
| 37 | 8p22 | 13207 | 13469 | 262 |
| 38 | 8p22 | 17007 | 17111 | 104 |
| 39 | 8p22 | 17636 | 17766 | 130 |
| 40 | 8p22 | 17895 | 18123 | 228 |
| 41 | 8p21.3 | 19855 | 20050 | 195 |
| 42 | 8p21.3 | 22202 | 22355 | 153 |
| 43 | 8p21.3 | 22954 | 23015 | 61 |
| 44 | 8p21.3 | 23105 | 23117 | 12 |
| 45 | 8p21.3-p21.2 | 23253 | 23367 | 114 |
| 46 | 8p21.2 | 23595 | 23762 | 167 |
| 47 | 8p21.2 | 24360 | 24440 | 80 |
| 48 | 8p21.2 | 25419 | 25945 | 526 |
| 49 | 8p21.1 | 27431 | 27458 | 27 |
| 50 | 8p21.1 | 27555 | 27661 | 106 |
| 51 | 8p21.1 | 28256 | 28260 | 4 |
| 52 | 8p21.1-p12 | 28701 | 29037 | 336 |
| 53 | 8p12 | 30043 | 30055 | 12 |
| 54 | 8p12 | 30157 | 30394 | 237 |
| 55 | 8p12 | 30524 | 30686 | 162 |
| 56 | 8p12 | 31150 | 31607 | 457 |
| 57 | 9p24.3 | 855 | 883 | 28 |
| 58 | 9p24.3 | 1147 | 2078 | 931 |
| 59 | 9p24.3-p24.2 | 2187 | 2525 | 338 |
| 60 | 9p24.2 | 2906 | 3718 | 812 |
| 61 | 9p24.2 | 3922 | 4488 | 566 |
| 62 | 9p24.2-9p24.1 | 4577 | 5410 | 833 |
| 63 | 9p24.1 | 5500 | 5764 | 264 |
| 64 | 9p24.1 | 6320 | 6516 | 196 |
| 65 | 9p24.1 | 6684 | 7160 | 476 |
| 66 | 9p24.1-p23 | 8508 | 9612 | 1104 |
| 67 | 9p23 | 10872 | 12038 | 1166 |
| 68 | 9p23 | 12656 | 12683 | 27 |
| 69 | 9p23 | 13207 | 13364 | 157 |
| 70 | 9p23 | 14015 | 14169 | 154 |
| 71 | 9p22.3 | 14387 | 14670 | 283 |
| 72 | 9p22.3 | 14877 | 15735 | 858 |
| 73 | 9p22.3 | 15865 | 16408 | 543 |
| 74 | 9p22.2 | 16650 | 17492 | 842 |
| 75 | 9p22.2 | 17699 | 18388 | 689 |
| 76 | 9p22.1 | 18629 | 18828 | 199 |
| 77 | 9p22.1 | 19012 | 19047 | 35 |
| 78 | 9p22.1 | 19203 | 19347 | 144 |
| 79 | 9p22.1 | 19501 | 19775 | 274 |
| 80 | 9p21.3 | 20111 | 20640 | 529 |
| 81 | 9p21.3 | 20810 | 23425 | 2615 |
| 82 | 9p21.3 | 25357 | 25573 | 216 |
| 83 | 9p21.2 | 26989 | 27052 | 63 |
| 84 | 9p21.1 | 28660 | 29012 | 352 |
| 85 | 9p21.1 | 29743 | 30134 | 391 |
| 86 | 9p21.1 | 33036 | 33114 | 78 |
| 87 | 9p13.3 | 33293 | 33337 | 44 |
| 88 | 9p13.3 | 33852 | 33890 | 38 |
| 89 | 9p13.3 | 34177 | 34300 | 123 |
| 90 | 9p13.3 | 34462 | 34601 | 139 |
| 91 | 9p13.2 | 36353 | 36390 | 37 |
| 92 | 9p13.2 | 37413 | 37491 | 78 |
| 93 | 9p13.2 | 37750 | 37768 | 18 |
| 94 | 9p13.1 | 39130 | 39277 | 147 |
| 95 | 9q21.11 | 70341 | 70534 | 193 |
| 96 | 9q21.11 | 70724 | 70838 | 114 |
| 97 | 9q21.11 | 71197 | 71372 | 175 |
| 98 | 9q21.11-q21.12 | 72158 | 72255 | 97 |
| 99 | 9q21.12 | 72430 | 72616 | 186 |
| 100 | 9q21.12-q21.13 | 73671 | 74134 | 463 |
| 101 | 9q21.13 | 74166 | 74352 | 186 |
| 102 | 9q21.13 | 74830 | 74975 | 145 |
| 103 | 9q21.13 | 75982 | 76491 | 509 |
| 104 | 9q21.13 | 76631 | 76874 | 243 |
| 105 | 9q21.13 | 77497 | 77650 | 153 |
| 106 | 9q21.13 | 77892 | 78291 | 399 |
| 107 | 9q21.13-q21.2 | 78628 | 79228 | 600 |
| 108 | 9q21.2 | 79672 | 79876 | 204 |
| 109 | 9q21.31 | 81746 | 81920 | 174 |
| 110 | 9q21.31 | 82612 | 82707 | 95 |
| 111 | 9q21.31-q21.32 | 83475 | 84694 | 1219 |
| 112 | 9q21.32 | 85546 | 85662 | 116 |
| 113 | 9q21.32 | 85760 | 86142 | 382 |
| 114 | 9q21.32 | 86512 | 86826 | 314 |
| 115 | 9q21.33 | 87357 | 87488 | 131 |
| 116 | 9q21.33 | 87779 | 87825 | 46 |
| 117 | 9q21.33 | 88037 | 88536 | 499 |
| 118 | 9q21.33 | 89290 | 89606 | 316 |
| 119 | 9q21.33 | 90220 | 90373 | 153 |
| 120 | 9q22.1 | 90764 | 90798 | 34 |
| 121 | 9q22.1 | 91153 | 91192 | 39 |
| 122 | 9q22.1-q22.2 | 91624 | 92604 | 980 |
| 123 | 9q22.2 | 93092 | 93525 | 433 |
| 124 | 9q22.31 | 94049 | 94095 | 46 |
| 125 | 9q22.31 | 94513 | 94542 | 29 |
| 126 | 9q22.31 | 95325 | 95393 | 68 |
| 127 | 9q22.31 | 96095 | 96248 | 153 |
| 128 | 9q22.31 | 96395 | 96426 | 31 |
| 129 | 9q22.32 | 96769 | 97699 | 930 |
| 130 | 9q22.32 | 97977 | 98263 | 286 |
| 131 | 9q22.32 | 98519 | 98834 | 315 |
| 132 | 9q22.33 | 99408 | 99653 | 245 |
| 133 | 9q22.33 | 99884 | 99962 | 78 |
| 134 | 9q22.33 | 101635 | 101709 | 74 |
| 135 | 9q22.33 | 101986 | 102246 | 260 |
| 136 | 9q22.33-q31.1 | 102379 | 102646 | 267 |
| 137 | 9q31.1 | 103035 | 103127 | 92 |
| 138 | 9q31.1 | 104542 | 105473 | 931 |
| 139 | 9q31.1 | 106688 | 107422 | 734 |
| 140 | 9q31.2 | 108203 | 108487 | 284 |
| 141 | 9q31.2 | 109132 | 109287 | 155 |
| 142 | 9q31.2 | 110712 | 110745 | 33 |
| 143 | 9q31.2-q31.3 | 111230 | 111522 | 292 |
| 144 | 9q31.3 | 112052 | 112225 | 173 |
| 145 | 9q31.3 | 112497 | 112676 | 179 |
| 146 | 9q31.3 | 113512 | 113578 | 66 |
| 147 | 9q31.3 | 113882 | 114207 | 325 |
| 148 | 9q31.3 | 114460 | 114686 | 226 |
| 149 | 9q32 | 115075 | 115081 | 6 |
| 150 | 9q32 | 115118 | 115172 | 54 |
| 151 | 9q32 | 115209 | 115249 | 40 |
| 152 | 9q32 | 116132 | 116591 | 459 |
| 153 | 9q32 | 116785 | 116822 | 37 |
| 154 | 9q32 | 117093 | 117716 | 623 |
| 155 | 9q33.1 | 118202 | 118204 | 2 |
| 156 | 9q33.1 | 118492 | 118881 | 389 |
| 157 | 9q33.1 | 119211 | 119488 | 277 |
| 158 | 9q33.1 | 120037 | 121126 | 1089 |
| 159 | 9q33.1-q33.2 | 122190 | 122586 | 396 |
| 160 | 9q33.2 | 122793 | 122836 | 43 |
| 161 | 9q33.2 | 124559 | 124682 | 123 |
| 162 | 9q33.2 | 124822 | 124855 | 33 |
| 163 | 9q33.2 | 124913 | 124984 | 71 |
| 164 | 9q33.2-q33.3 | 125762 | 125937 | 175 |
| 165 | 9q33.3 | 126479 | 126556 | 77 |
| 166 | 9q33.3 | 126746 | 126820 | 74 |
| 167 | 9q33.3 | 126982 | 127199 | 217 |
| 168 | 9q33.3 | 127547 | 127679 | 132 |
| 169 | 9q33.3 | 128143 | 128458 | 315 |
| 170 | 9q33.3 | 128611 | 128637 | 26 |
| 171 | 9q33.3 | 129209 | 129281 | 72 |
| 172 | 9q33.3 | 129357 | 129482 | 125 |
| 173 | 9q33.3 | 129592 | 129606 | 14 |
| 174 | 9q33.3 | 129963 | 130024 | 61 |
| 175 | 9q33.3-9q34.11 | 130234 | 130346 | 112 |
| 176 | 9q34.11 | 130495 | 130508 | 13 |
| 177 | 9q34.11 | 130635 | 130673 | 38 |
| 178 | 9q34.11 | 130907 | 131027 | 120 |
| 179 | 9q34.11 | 131436 | 131438 | 2 |
| 180 | 9q34.11 | 131524 | 131549 | 25 |
| 181 | 9q34.11 | 131652 | 132287 | 635 |
| 182 | 9q34.11 | 132990 | 133060 | 70 |
| 183 | 9q34.11 | 133327 | 133443 | 116 |
| 184 | 9q34.13 | 134127 | 134151 | 24 |
| 185 | 9q34.13 | 134251 | 134459 | 208 |
| 186 | 9q34.13 | 134711 | 134803 | 92 |
| 187 | 9q34.13 | 135396 | 135528 | 132 |
| 188 | 9q34.3 | 139284 | 139338 | 54 |
| 189 | 9q34.3 | 139777 | 139853 | 76 |
| 190 | 10p12.1 | 27334 | 27353 | 19 |
| 191 | 10q21.2 | 63668 | 63823 | 155 |
| 192 | 10q21.3 | 69317 | 69418 | 101 |
| 193 | 10q22.2 | 75472 | 75567 | 95 |
| 194 | 10q23.1 | 83990 | 84685 | 695 |
| 195 | 10q23.2 | 88618 | 88673 | 55 |
| 196 | 10q23.2 | 89302 | 89412 | 110 |
| 197 | 10q23.31 | 89791 | 90226 | 435 |
| 198 | 10q23.31 | 91473 | 91524 | 51 |
| 199 | 10q23.31 | 92085 | 92353 | 268 |
| 200 | 10q23.32 | 93031 | 93194 | 163 |
| 201 | 10q23.32 | 93580 | 93614 | 34 |
| 202 | 10q23.33 | 94204 | 94505 | 301 |
| 203 | 10q23.33 | 96034 | 96351 | 317 |
| 204 | 10q24.1 | 97479 | 97548 | 69 |
| 205 | 10q24.2 | 101360 | 101365 | 5 |
| 206 | 10q24.31 | 101968 | 101982 | 14 |
| 207 | 10q24.31 | 102298 | 102418 | 120 |
| 208 | 10q24.32 | 103128 | 103166 | 38 |
| 209 | 10q24.32 | 103817 | 103884 | 67 |
| 210 | 10q24.33 | 105052 | 105174 | 122 |
| 211 | 10q25.1 | 106041 | 106049 | 8 |
| 212 | 10q25.1 | 109744 | 110719 | 975 |
| 213 | 10q25.1-q25.2 | 111850 | 112002 | 152 |
| 214 | 10q25.2-q25.3 | 114864 | 115302 | 438 |
| 215 | 10q25.3 | 118462 | 118656 | 194 |
| 216 | 10q26.11 | 121102 | 121189 | 87 |
| 217 | 10q26.11 | 121420 | 121448 | 28 |
| 218 | 10q26.11 | 121561 | 121597 | 36 |
| 219 | 10q26.13 | 123628 | 123712 | 84 |
| 220 | 10q26.13 | 126507 | 126571 | 64 |
| 221 | 10q26.2 | 128731 | 128806 | 75 |
| 222 | 10q26.3 | 132937 | 132997 | 60 |
| 223 | 10q26.3 | 133635 | 133644 | 9 |
| 224 | 11p15.4 | 6822 | 6958 | 136 |
| 225 | 11p15.4 | 7774 | 7850 | 76 |
| 226 | 11p15.2 | 14665 | 14837 | 172 |
| 227 | 11p15.1 | 18492 | 18541 | 49 |
| 228 | 11p14.1 | 30210 | 30309 | 99 |
| 229 | 11p11.2 | 47664 | 47682 | 18 |
| 230 | 11q22.1 | 97992 | 99195 | 1203 |
| 231 | 11q22.2 | 102611 | 102629 | 18 |
| 232 | 11q22.3 | 104865 | 105016 | 151 |
| 233 | 11q22.3 | 107235 | 107512 | 277 |
| 234 | 11q22.3 | 107761 | 107848 | 87 |
| 235 | 11q23.1 | 111400 | 111421 | 21 |
| 236 | 11q23.3 | 114590 | 114646 | 56 |
| 237 | 11q23.3 | 117844 | 117865 | 21 |
| 238 | 11q24.3 | 128566 | 128761 | 195 |
| 239 | 11q24.3 | 129572 | 129603 | 31 |
| 240 | 13q11 | 19254 | 19317 | 63 |
| 241 | 13q14.11 | 44867 | 44991 | 124 |
| 242 | 13q14.2 | 48923 | 48964 | 41 |
| 243 | 14q31.1 | 79591 | 79735 | 144 |
| 244 | 15q13.1 | 29142 | 29214 | 72 |
| 245 | 15q14 | 39466 | 39480 | 14 |
| 246 | 16p13.11 | 14956 | 14988 | 32 |
| 247 | 16q22.1 | 68723 | 68751 | 28 |
| 248 | 16q24.1 | 85997 | 86024 | 27 |
| 249 | 17p13.3 | 351 | 549 | 198 |
| 250 | 17p13.3 | 609 | 621 | 12 |
| 251 | 17p13.3 | 933 | 955 | 22 |
| 252 | 17p13.3 | 1059 | 1105 | 46 |
| 253 | 17p13.3 | 1279 | 1287 | 8 |
| 254 | 17p13.3 | 1391 | 1439 | 48 |
| 255 | 17p13.3 | 1613 | 1703 | 90 |
| 256 | 17p13.3 | 2976 | 3065 | 89 |
| 257 | 17p13.3-p13.2 | 3269 | 3466 | 197 |
| 258 | 17p13.2 | 5260 | 5279 | 19 |
| 259 | 17p13.1 | 9114 | 9263 | 149 |
| 260 | 17p13.1 | 9537 | 9701 | 164 |
| 261 | 17p12 | 11089 | 11665 | 576 |
| 262 | 17p12 | 11990 | 12428 | 438 |
| 263 | 17p12 | 12639 | 12854 | 215 |
| 264 | 17p12 | 13498 | 13587 | 89 |
| 265 | 17p12 | 14052 | 14277 | 225 |
| 266 | 17p12 | 15871 | 15883 | 12 |
| 267 | 17p11.2 | 16132 | 16186 | 54 |
| 268 | 17p11.2 | 17111 | 17155 | 44 |
| 269 | 17p11.2 | 18612 | 18647 | 35 |
| 270 | 17p11.2 | 19624 | 19715 | 91 |
| 271 | 17q21.31 | 41587 | 41700 | 113 |
| 272 | 17q21.33 | 48801 | 48882 | 81 |
| 273 | 17q22 | 55117 | 55129 | 12 |
| 274 | 18p11.21 | 12421 | 12578 | 157 |
| 275 | 18q22.1 | 61718 | 62008 | 290 |
| 276 | 19p13.3 | 2764 | 2785 | 21 |
| 277 | 21q21.3 | 28368 | 28418 | 50 |
| 278 | 22q11.22 | 22681 | 22712 | 31 |
| 279 | 22q13.1 | 37689 | 37715 | 26 |

| **Supplementary table 3:** Correlations between identified CNVs in urine and clinicopathological characteristics of 123 BC patients [median (interquartile range)] | | | | | | | |
| --- | --- | --- | --- | --- | --- | --- | --- |
| **Parameters** | **BC urine samples detected by real-time PCR (n=123)** | | | | | | |
|  | **Cases** | **CEP63** | **p value** | **FOSL2** | **p value** | **PAQR6** | **p value** |
| **Age** |  |  | 0.302 |  | 0.123 |  | 0.761 |
| ≤ 65 | 73 | 0.867(0.564-1.624) |  | 1.174(1.057-1.302) |  | 1.363(1.072-1.569) |  |
| > 65 | 50 | 1.079(0.639-1.773) |  | 1.226(1.135-1.328) |  | 1.388(1.022-1.561) |  |
| **Sex** |  |  | 0.780 |  | 0.135 |  | 0.591 |
| Male | 84 | 1.026(0.606-1.555) |  | 1.178(1.065-1.307) |  | 1.347(1.057-1.531) |  |
| Female | 39 | 1.075(0.578-1.676) |  | 1.246(1.123-1.348) |  | 1.444(1.069-1.606) |  |
| **Stage** |  |  | 0.465 |  | 0.068 |  | 0.950 |
| Ta–T1 | 104 | 1.031(0.58-1.631) |  | 1.18(1.075-1.283) |  | 1.394(1.065-1.583) |  |
| T2–T4 | 19 | 1.121(0.734-2.493) |  | 1.314(1.123-1.444) |  | 1.359(1.026-1.533) |  |
| **Histologic grade** |  |  | 0.139 |  | 0.051 |  | 0.989 |
| Low grade | 78 | 1.024(0.576-1.505) |  | 1.173(1.071-1.279) |  | 1.393(1.072-1.583) |  |
| High grade | 45 | 1.169(0.652-2.937) |  | 1.266(1.115-1.384) |  | 1.359(1.04-1.539) |  |

| **Supplementary table 4**. Correlation between identified CNVs in urine and clinicopathological characteristics of 40 BC patients [median  (interquartile range)] | | | | | | | | |
| --- | --- | --- | --- | --- | --- | --- | --- | --- |
| **Parameters** | **BC urine samples detected by 3D digital PCR (n=40)** | | | | | | |  |
|  | **Cases** | **CEP63** | **p value** | **FOSL2** | **p value** | **PAQR6** | **p value** |  |
| **Age** |  |  | 0.386 |  | 0.598 |  | 0.807 |  |
| ≤ 65 | 20 | 1.003(0.959-1.106) |  | 1.015(0.902-1.143) |  | 0.999(0.892-1.121) |  |  |
| > 65 | 20 | 0.978(0.882-1.070) |  | 1.052(0.957-1.111) |  | 1.027(0.897-1.136) |  |  |
| **Sex** |  |  | 0.554 |  | 0.388 |  | 0.964 |  |
| Male | 29 | 1.007(0.946-1.108) |  | 1.047(0.958-1.137) |  | 1.037(0.876-1.126) |  |  |
| Female | 11 | 0.975(0.946-1.078) |  | 0.926(0.897-1.115) |  | 0.975(0.909-1.143) |  |  |
| **Stage** |  |  | **2.81E-04** |  | **0.046** |  | 0.058 |  |
| Ta–T1 | 21 | 0.957(0.865-0.990) |  | 0.958(0.904-1.078) |  | 0.950(0.896-1.041) |  |  |
| T2–T4 | 19 | 1.046(1.016-1.173) |  | 1.076(0.979-1.165) |  | 1.073(0.864-1.194) |  |  |
| **Histologic grade** |  |  | **0.042** |  | 0.473 |  | 0.684 |  |
| Low grade | 19 | 0.958(0.925-1.007) |  | 0.979(0.926-1.081) |  | 0.975(0.909-1.045) |  |  |
| High grade | 21 | 1.035(0.978-1.132) |  | 1.074(0.914-1.157) |  | 1.050(0.850-1.151) |  |  |
